# Alanine supplementation exploits glutamine dependency induced by SMARCA4/2-loss

**DOI:** 10.1038/s41467-023-38594-3

**Published:** 2023-05-20

**Authors:** Xianbing Zhu, Zheng Fu, Shary Y. Chen, Dionzie Ong, Giulio Aceto, Rebecca Ho, Jutta Steinberger, Anie Monast, Virginie Pilon, Eunice Li, Monica Ta, Kyle Ching, Bianca N. Adams, Gian L. Negri, Luc Choiniere, Lili Fu, Kitty Pavlakis, Patrick Pirrotte, Daina Z. Avizonis, Jeffrey Trent, Bernard E. Weissman, Ramon I. Klein Geltink, Gregg B. Morin, Morag Park, David G. Huntsman, William D. Foulkes, Yemin Wang, Sidong Huang

**Affiliations:** 1 Department of Biochemistry, McGill University, Montreal, QC Canada; 2 Rosalind & Morris Goodman Cancer Institute, McGill University, Montreal, QC Canada; 3 Department of Pathology and Laboratory Medicine, University of British Columbia, Vancouver, BC Canada; 4 Department of Molecular Oncology, British Columbia Cancer Research Institute, Vancouver, BC Canada; 5 Canada’s Michael Smith Genome Science Centre, British Columbia Cancer Research Institute, Vancouver, BC Canada; 6 Rosalind & Morris Goodman Cancer Institute, Metabolomics Innovation Resource, McGill University, Montreal, QC Canada; 7 Department of Pathology, McGill University Health Centre, Montreal, QC Canada; 8 Department of Pathology, IASO women’s hospital, Athens, Greece; 9 Cancer & Cell Biology Division, Translational Genomics Research Institute, Phoenix, AZ USA; 10 Integrated Mass Spectrometry Shared Resource, City of Hope Comprehensive Cancer Center, Duarte, CA USA; 11 Translational Genomics Research Institute, Division of Integrated Cancer Genomics, Phoenix, AZ USA; 12 Department of Pathology and Laboratory Medicine, University of North Carolina, Chapel Hill, NC USA; 13 Lineberger Comprehensive Cancer Center, University of North Carolina, Chapel Hill, NC USA; 14 Department of Medical Genetics, University of British Columbia, Vancouver, BC Canada; 15 Department of Obstetrics and Gynaecology, University of British Columbia, Vancouver, BC Canada; 16 Departments of Human Genetics, Medicine and Oncology McGill University, Montreal, QC Canada; 17 Division of Medical Genetics, Department of Specialized Medicine and Cancer Research Program, McGill University Health Centre, Montreal, QC Canada; 18 Division of Medical Genetics, Department of Specialized Medicine and Lady Davis Institute, Jewish General Hospital, McGill University, Montreal, QC Canada

**Keywords:** Molecular biology, Cancer metabolism, Targeted therapies

## Abstract

SMARCA4 (BRG1) and SMARCA2 (BRM) are the two paralogous ATPases of the SWI/SNF chromatin remodeling complexes frequently inactivated in cancers. Cells deficient in either ATPase have been shown to depend on the remaining counterpart for survival. Contrary to this paralog synthetic lethality, concomitant loss of SMARCA4/2 occurs in a subset of cancers associated with very poor outcomes. Here, we uncover that SMARCA4/2-loss represses expression of the glucose transporter GLUT1, causing reduced glucose uptake and glycolysis accompanied with increased dependency on oxidative phosphorylation (OXPHOS); adapting to this, these SMARCA4/2-deficient cells rely on elevated SLC38A2, an amino acid transporter, to increase glutamine import for fueling OXPHOS. Consequently, SMARCA4/2-deficient cells and tumors are highly sensitive to inhibitors targeting OXPHOS or glutamine metabolism. Furthermore, supplementation of alanine, also imported by SLC38A2, restricts glutamine uptake through competition and selectively induces death in SMARCA4/2-deficient cancer cells. At a clinically relevant dose, alanine supplementation synergizes with OXPHOS inhibition or conventional chemotherapy eliciting marked antitumor activity in patient-derived xenografts. Our findings reveal multiple druggable vulnerabilities of SMARCA4/2-loss exploiting a GLUT1/SLC38A2-mediated metabolic shift. Particularly, unlike dietary deprivation approaches, alanine supplementation can be readily applied to current regimens for better treatment of these aggressive cancers.

## Abstract (web summary)

Cancers with concomitant SMARCA4/2 deficiencies are often resistant to chemotherapies and confer a poor prognosis. Here, the authors identify a metabolic dependency of these tumours on glutamine as an energy source and, using multiple approaches, demonstrate the efficacy of therapeutically targeting this vulnerability.

## Introduction

The SWI/SNF family of ATP-dependent chromatin remodeling complexes regulate transcription by controlling chromatin accessibility^1,2^. Mutations affecting various SWI/SNF subunits are found in ~25% of all human cancers, highlighting their critical roles in tumorigenesis^3^. *SMARCA4*, which encodes one of the two mutually exclusive ATPases of the SWI/SNF complexes, is frequently inactivated by mutations; in contrast, *SMARCA2*, the paralog of *SMARCA4*, is rarely mutated but often epigenetically silenced in tumors^3,4^. Cancer cells deficient in either SMARCA4 or SMARCA2 are vulnerable to genetic ablation of the remaining paralog^5–8^, a synthetic lethality that is being explored as a potential therapeutic strategy. However, concurrent loss of both SMARCA4 and SMARCA2 (referred to as SMARCA4/2 thereafter) characterizes small cell carcinoma of the ovary, hypercalcemic type (SCCOHT), a rare but lethal ovarian cancer affecting young women without other recurrent genetic alterations^9–14^ and also occurs in other highly aggressive human malignancies, including non-small cell lung cancers (NSCLCs)^15,16^, undifferentiated thoracic sarcoma^17,18^, undifferentiated uterine sarcoma^19^, and dedifferentiated/undifferentiated carcinoma of various organs^20–23^. These clinical observations suggest that complete inactivation of SWI/SNF ATPase activity may promote cancer development and/or progression in these contexts.

SMARCA4/2-deficient cancers remain difficult to treat as they rarely harbor known druggable mutations and are resistant to conventional chemotherapies^24–26^. Recently, we and others have demonstrated that SMARCA4/2-deficient ovarian cancers rely on epigenetic reprogramming and are responsive to inhibitors targeting the polycomb repressive complex 2, histone deacetylases, and the bromodomain-containing protein 4^27–30^. We also uncovered that these cancer cells are sensitive to drugs targeting receptor tyrosine kinases^31^, CDK4/6^32,33^, and arginine^34^. Despite these encouraging findings, the clinical activities of these inhibitors remain unknown as they mostly suppress proliferation and may not completely eradicate SMARCA4/2-deficient cancer cells. Thus, it is important to identify additional therapeutic vulnerabilities in these cancers for developing the most effective treatment strategies.

In the present study, we uncover that SMARCA4/2 loss triggers a metabolic shift in cancer cells, resulting in a preference for utilizing glutamine over glucose as the primary carbon source to fuel the tricarboxylic acid (TCA) cycle and sustain oxidative phosphorylation (OXPHOS). Consequently, SMARCA4/2-deficient cancer cells and tumors are highly sensitive to clinically available inhibitors targeting OXPHOS or glutamine metabolism as well as supplementation of alanine that competes with glutamine import, providing multiple potential effective treatment options for targeting these hard-to-treat malignancies.

## Results

### SMARCA4/2-deficient cancer cells have reduced glycolysis and depend on OXPHOS

**Fig. 1:**
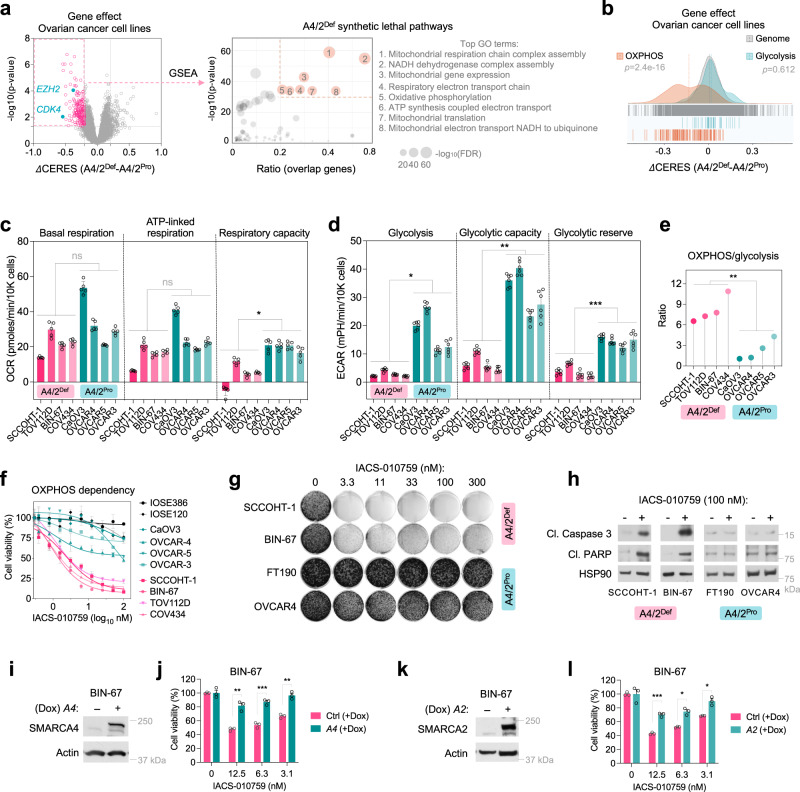
*SMARCA4/2*-loss in ovarian cancer cells results in impaired glycolysis and increased dependence on OXPHOS. **a** Left, a volcano plot showing the differential dependency of genes between *SMARCA4/2*-deficient (*A4/2*^Def^; *n* = 5) and proficient (*A4/2*^Pro^; *n* = 32) ovarian cancer cell lines, using the CERES gene effect data from DepMap genome-wide CRISPR-knockout screens (two-tailed t-test). Each dot denotes a gene. Genes whose deletion selectively impairs the growth of *SMARCA4/2*-deficient cancer cells (in red) were subjected to Gene Set Enrichment Analysis (GSEA). Right, dot plot reflecting the GO analysis of the top 200 hits essential for *SMARCA4/2*-deficient cancer cells. **b** Density plot showing the differential dependency of indicated gene groups between *SMARCA4/2*-deficient and proficient ovarian cancer cell lines as described in (**a**). Each line denotes a gene. *p*, *p* value; Kolmogorov Smirnov tests for OXPHOS or glycolysis genes compared to the whole genome. **c** Quantification of basal respiration (*p* = 0.1905), maximum respiratory capacity (*p* = 0.1213) and ATP-linked respiration (*p* = 0.0156) in indicated cell lines measured by Seahorse Mito Stress Test assay (two-tailed t-test, *n* = 5 independent experiments). **d** Quantification of basal glycolysis (*p* = 0.0222), glycolysis capacity (*p* = 0.0036) and glycolytic reserve (*p* = 0.0002) in indicated cell lines measured by Seahorse glycolysis stress test (two-tailed t-test, *n*  =  6 independent experiments). **e** Ratio of basal respiration to glycolysis between *A4/2*^Def^ vs *A4/2*^Pro^ ovarian cancer cell lines measured by Seahorse assays (two-tailed t-test, *p* = 0.0035). **f** Cell viability analyzes of the indicated cell lines treated with IACS-010759 for 3 days. *n*  =  3 independent experiments. **g** Colony-formation assay of cell lines treated with IACS-010759 for 10–15 days. **h** Immunoblots of cell lines treated with IACS-010759 for 3 days. Cl., cleaved. **i**–**l** Immunoblots (**i,** **k**) and cell viabilities (**j**, **l**) of BIN-67 cells, -/+ doxycycline (Dox)-inducible SMARCA4 (**i**, **j**) or SMARCA2 (**k**, **l**) re-expression, treated with IACS-010759 for 5 days. Two-tailed t-test, *n*  =  3 independent experiments. *p* values: **j** 0.0079, 0.0004, 0.0031; **l** 0.0006, 0.0131, 0.0249. *p < 0.05, **p < 0.01, ***p < 0.001. ns, not significant. Error bars, mean ± SD.

To systematically uncover genetic dependencies of SMARCA4/2-loss, we first focused on cell models of SCCOHT due to their simple genome^35^. We analyzed the genome-wide CRISPR/Cas9 knockout screens from the Cancer Dependency Map (DepMap, https://depmap.org) across 37 ovarian cancer cell lines (Supplementary Data 1): five are SMARCA4/2-deficient, including three SCCOHT cell lines (BIN-67, SCCOHT-1, COV434)^13,36^ and two dedifferentiated ovarian cancer cell lines (TOV-112D, OVK18)^29,36^, while the remaining 32 are SMARCA4/2-proficient^29,37^. Pathway enrichment analysis of the top 200 genes whose knockout was selectively lethal to SMARCA4/2-deficient cells revealed that the 8 highest ranked Gene Ontology (GO) terms are all related to mitochondria function and mostly involved in OXPHOS (Fig. 1a, Supplementary Data 2). We further examined the essentiality scores of all GO genes for OXPHOS and glycolysis, two major cellular energy suppliers. Unlike glycolysis genes that followed the same distribution of the whole genome, a greater portion of OXPHOS genes were more essential to SMARCA4/2-deficient cells (Fig. 1b). These unbiased observations suggest that SMARCA4/2-deficient ovarian cancer cells may rely on OXPHOS more than glycolysis for energy supply.

*SMARCA4*-mutant NSCLC cells with intact SMARCA2 depend on their elevated OXPHOS activity^38^. We profiled OXPHOS and glycolysis capacities in SMARCA4/2-deficient ovarian cancer cells (BIN-67, SCCOHT-1, COV434, TOV-112D) and SMARCA4/2-proficient high-grade serous ovarian carcinoma (HGSOC) cells (OVCAR3, OVCAR4, OVCAR5, CaOV3). However, we found that neither basal nor ATP-linked respiration rates of SMARCA4/2-deficient cells differed significantly from controls; SMARCA4/2-deficient cells even had lower maximum respiratory capacity (Fig. 1c, Supplementary Fig. 1a); in contrast, glycolysis, glycolytic capacity, and glycolytic reserve of SMARCA4/2-deficient cells were all significantly lower compared to controls (Fig. 1d, Supplementary Fig. 1b). Consequently, OXPHOS/glycolysis ratios were significantly elevated in SMARCA4/2-deficient cells compared to the proficient controls (Fig. 1e). Therefore, unlike *SMARCA4*-mutant NSCLC cells with intact SMARCA2^38^, the OXPHOS dependency in SMARCA4/2-deficient ovarian cancer cells is not due to increased OXPHOS activity, but rather attributed to repressed glycolysis.

Validating the above findings, SMARCA4/2-deficient ovarian cancer cells, but not proficient controls including HGSOC and non-transformed ovarian or fallopian tube epithelial cells (IOSE120, IOSE386, FT190), were highly sensitive to IACS-010759, a selective inhibitor of electron transport chain Complex I^39,40^, in both viability (Fig. 1f) and growth (Fig. 1g) assays, accompanied with a strong apoptotic response (Fig. 1h, Supplementary Fig. 1c). Similar results were also obtained using inhibitors targeting Complex I (rotenone, metformin, phenformin), Complex III (antimycin A), and ATPase (oligomycin) (Supplementary Fig. 1d–g). While the full restoration of SMARCA4/2 suppresses SCCOHT proliferation^13,32^, low levels of their re-expression in SCCOHT cells using a doxycycline inducible system conferred resistance to IACS-010759 (Fig. 1i–l, Supplementary Fig. 1h, i). Conversely, CRISPR/Cas9-mediated *SMARCA4* knockout in FT190 cells partially sensitized them to IACS-010759 and *SMARCA2* knockdown using two independent shRNAs in these *SMARCA4* knockout cells further increased their drug sensitivities (Supplementary Fig. 1j, k). Together, these data establish that SMARCA4/2-deficient ovarian cancer cells exhibit reduced glycolysis and highly depend on OXPHOS.

### SMARCA4/2-loss represses GLUT1 expression leading to impaired glucose uptake

**Fig. 2:**
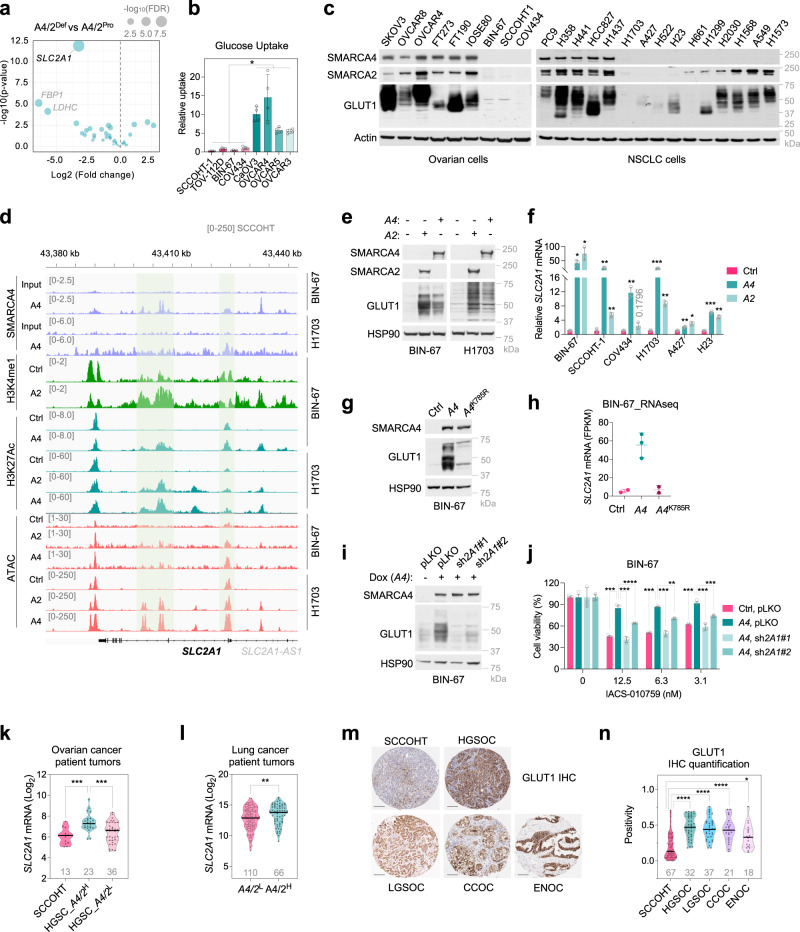
*SMARCA4/2*-loss causes *GLUT1* deficiency contributing to OXPHOS dependency. **a** Volcano plot of differentially expressed glycolysis genes between *SMARCA4/2*-deficient (*A4/2*^Def^) and proficient (*A4/2*^Pro^) ovarian cancer cell lines described in Fig. 1a. Each dot represents a gene. Wald test (DEseq2). **b** Cell line glucose uptake capacity. Two-tailed t-test, *n*  =  4 independent experiments, *p* values = 0.0286. **c** Immunoblots. **d** Representative browser track of ChIP-Seq and ATAC-Seq on the *SLC2A1* locus in indicated cells ± SMARCA4/2^33,42,43^. **e** Immunoblots of cell lines ± *SMARCA4/2* restoration. **f** Relative *SLC2A1* mRNA in cell lines ± *SMARCA4/2* restoration by RT-qPCR. One-way ANOVA corrected for multiple comparisons, *n*  =  3 independent experiments. *p* values: BIN-67, 0.0386, 0.0459; SCCOHT-1, 0.0084, 0.0017; COV434, 0.0093, 0.1796; H1703, 0.0006, 0.0028; A427, 0.0022, 0.0201; H23, 0.0008, 0.0058. **g** Immunoblots of BIN-67 ± ectopic expression of wild type or an ATPase dead mutant (K785R) of SMARCA4. **h** *SCL2A1* mRNA abundance (FPKM) in BIN-67 ± ectopic expression of SMARCA4 wild type or K785R obtained from GSE117311. **i** Immunoblots of BIN-67 expressing doxycycline (Dox) inducible *SMARCA4* ± pLKO control or shRNAs targeting *SLC2A1* (sh*2A1*). **j** Cell viability of the cells described in (**i**) treated with IACS-010759 for 5 days. One-way ANOVA corrected for multiple comparisons, *n*  =  3 independent experiments. *p* values: pLKO vs sh*2A1*#2 (12.5), 0.0006, pLKO vs sh*2A1*#2 (3.1), 0.0007, others <0.0001. **k**, **l** Violin plot showing *SLC2A1* mRNA levels in SCCOHT, HGSOC (**k**) and lung cancer (**l**) patient tumors stratified based on *SMARCA4/2* expression by quartile. H: high; L: low. Left, one-way ANOVA corrected for multiple comparisons, *p* values: 0.0001, 0.0007; right, two-tailed t-test, *p* value: 0.0086. **m**, **n** Representative images (**m**) and quantification (**n**) of GLUT1 immunohistochemistry (IHC) analysis of SCCOHT patient tumors compared to other ovarian carcinoma subtypes. HGSOC, high-grade serous ovarian carcinoma, LGSOC, low-grade serous ovarian carcinoma, CCOC, clear cell ovarian carcinoma, ENOC, endometrioid ovarian carcinoma. Scale bar: 100 μm. One-way ANOVA corrected for multiple comparisons (ENOC – 0.0208, others <0.0001). Patient numbers are indicated in grey below each group. *p < 0.05, **p < 0.01, ***p < 0.001, ****p < 0.0001. Error bars, mean ± SD.

To determine the mechanism underlying repressed glycolysis in SMARCA4/2-deficient cancer cells, we analyzed mRNA expression of genes involved in glycolysis and glucose metabolism-related pathways using RNA-seq data, available from DepMap, for the ovarian cell lines used in the above knockout screen analysis. *SLC2A1*, encoding the key glucose transporter GLUT1^41^, was the top ranked gene whose expression was significantly lower in SMARCA4/2-deficient cells compared to proficient controls (Fig. 2a, Supplementary Data 3) that was validated by quantitative RT-PCR (Supplementary Fig. 2a). Accordingly, glucose uptake capacities of SMARCA4/2-deficient ovarian cancer cells were strongly reduced (Fig. 2b), consistent with their markedly low GLUT1 protein abundance compared to controls (Fig. 2c, left, Supplementary Fig. 2b); ectopic expression of GLUT1 in SMARCA4/2-deficient cells significantly increased glucose uptake (Supplementary Fig. 2c, d). These findings suggest that reduced GLUT1 expression limits glucose uptake and glycolysis in SMARCA4/2-deficient cancer.

Given that SMARCA4/2-loss also occurs in NSCLC, we examined the correlation between SMARCA4/2 and GLUT1 expression in a NSCLC cell line panel. Indeed, SMARCA4/2-deficient NSCLC cells expressed the lowest levels of GLUT1 while SMARCA4-deficient cells with intact SMARCA2 expressed intermediate levels of GLUT1 (Fig. 2c, right), suggesting the redundancy of SMARCA4/2 in promoting GLUT1 expression. This is supported by our observations in transcriptomic datasets of SMARCA4/2-deficient BIN-67 (SCCOHT)^42^ and A427 (NSCLC)^27,43^ cells ± restoration of SMARCA4 or SMARCA2 and in SMARCA4-deficient H1944 (NSCLC)^44^ cells ± SMARCA2 knockdown: *SLC2A1* was the only gene related to glucose transport or metabolism that was consistently activated by SMARCA4/2 among these data sets (Supplementary Fig. 2e, f, Supplementary Data 4). Furthermore, in ChIP-seq datasets of BIN-67^42,43^ and H1703^33^ cells upon SMARCA4-restoration, we also observed strong SMARCA4 occupancy at the promoter, gene body, and distal sites of the *SLC2A1* locus, where elevated H3K27Ac and H3K4me1 ChIP-seq peaks (active enhancer marks) and the ATAC-seq peaks (chromatin accessibility) were also detected in these cells upon re-expression of SMARCA4 or SMARCA2 (Fig. 2d). These data indicate that SMARCA4/2 promote GLUT1 expression by directly activating *SLC2A1* transcription. Validating this, re-expression of SMARCA4 or SMARCA2 in SCCOHT and NSCLC cells upregulated *SLC2A1*/GLUT1 expression (Fig. 2e, f, Supplementary Fig. 2g), accompanied with elevated glucose uptake (Supplementary Fig 2h), whereas ectopic expression of the ATPase dead mutant variant (K785R) of SMARCA4 failed to upregulate GLUT1 protein (Fig. 2g) and mRNA^42^ (Fig. 2h). Conversely, suppression of *SMARCA4/2* in FT190 cells decreased their GLUT1 expression levels (Supplementary Fig. 2i). Together, these results establish that SMARCA4/2 loss directly reduces the transcription of *SLC2A1*, resulting in GLUT1 deficiency and subsequent impaired glucose uptake in these cancer cells.

### GLUT1 deficiency contributes to the OXPHOS dependency in SMARCA4/2-deficient cancer cells

It is well-recognized that cancer cells depend on OXPHOS upon glucose limitation^45^. In line with this, addition of BAY-876, a selective GLUT1 inhibitor^46^, synergized with IACS-010759 in suppressing SMARCA4/2-proficient OVCAR4 and H1437 cells (Supplementary Fig. 2j). In contrast, GLUT1 inhibition did not further enhance IACS-010759 responses in SMARCA4/2-deficient SCCOHT-1 and H1703 cells (Supplementary Fig. 2j), which are already highly sensitive to OXPOHS inhibition alone compared to SMARCA4/2-proficient controls (Fig. 1f, g), suggesting that the GLUT1 deficiency caused by SMARCA4/2 loss may underline the hypersensitivity of SMARCA4/2-deficient cells to OXPHOS inhibition. Supporting this, while SMARCA4 restoration in BIN-67 (Fig. 2i, j) and H1703 cells (Supplementary Fig. 2k, l) conferred resistance to IACS-010759, blocking GLUT1 induction caused by SMARCA4 re-expression using two independent shRNAs in these cells re-sensitized them to OXPHOS inhibition. However, even though ectopic expression of GLUT1 in COV434, BIN-67 and H1703 cells restored glucose uptake (Supplementary Fig. 2c, d, m, n), this only caused partial resistance to OXPHOS inhibition by IACS-010759 in COV434 but not in BIN-67 and H1703 cells (Supplementary Fig. 2o). This is likely to due to that SMARCA4/2 loss may impact other nodes of the glycolysis pathway in addition to causing GLUT1 deficiency, which can be cell line specific and remains to be further investigated. Notably, we previously found that SMARCA4/2-loss causes reduced calcium ion (Ca^2+^) transfer from the endoplasmic reticulum to mitochondria^26^. This defective Ca^2+^ transfer may also contribute to the suppression of glycolysis since several key glycolytic enzymes such as glyceraldehyde-3-phosphate dehydrogenase (GAPDH) and phosphofructokinase (PFK) are known to be activated by Ca^2+^/calmodulin^47,48^; this requires further investigations. Nevertheless, our data support that the GLUT1 deficiency induced by SMARCA4/2 loss is a key contributor to the OXPHOS dependency in these SMARCA4/2-deficient cancer cells.

### GLUT1 expression is reduced in SMARCA4/2-deficient cancers

Extending on our above findings, in DepMap RNA-seq datasets of ovarian and lung cancer cell line panels, the expression of *SLC2A1* mRNA significantly and positively correlated with that of *SMARCA2* and *SMARCA4* in cells with low (bottom quartile) *SMARCA4* or *SMARCA2* expression, respectively (Supplementary Fig. 2p, q). Similar results were obtained by analyzing the DepMap Reverse Phase Protein Array data of these cell lines (Supplementary Fig. 2r). Furthermore, analysis of the RNA-seq datasets of SCCOHT^26^ and TCGA HGSOC^49^ showed that SCCOHT or HGSOCs with low *SMARCA4/2* expressed significantly lower *SLC2A1* than HGSOCs with high *SMARCA4/2* (Fig. 2k). A similar correlation was also observed in lung cancer patient tumors (cBioportal) (Fig. 2l). Using immunohistochemistry, we further confirmed that SCCOHT tumors expressed significantly lower levels of GLUT1 than four other ovarian cancer subtypes (Fig. 2m, n). Together, our findings show that GLUT1 downregulation is a unique feature of SMARCA4/2-deficient cancers.

### SMARCA4/2-deficient cancer cells utilize glutamine to sustain the TCA cycle

**Fig. 3:**
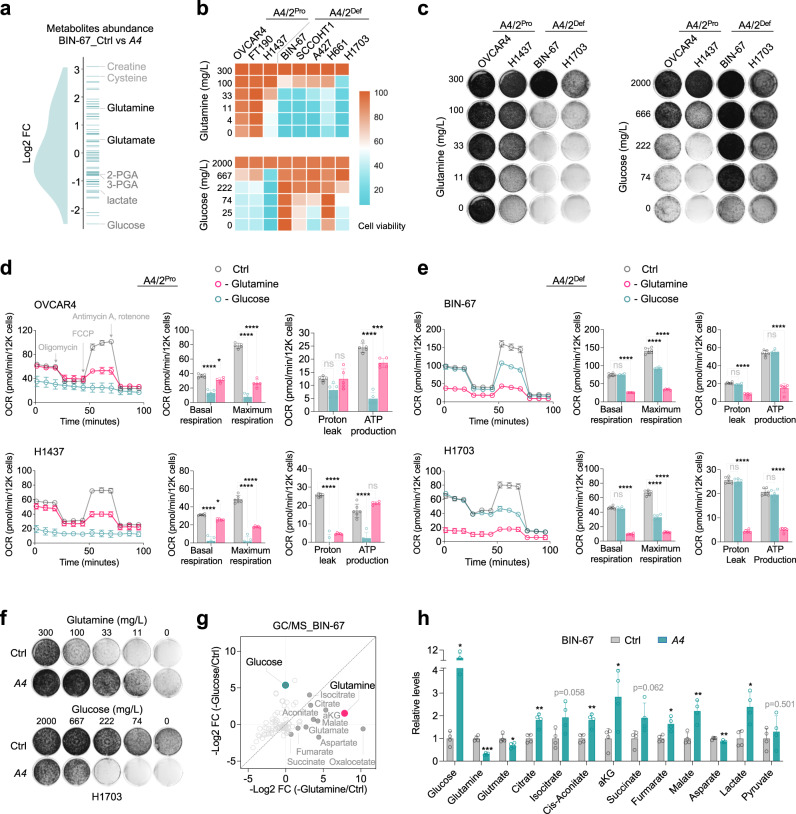
SMARCA4/2 loss shifts cancer cells to use glutamine as a carbon source for energy supply. **a** Density plot showing 70 metabolites related to the TCA cycle in BIN-67 cells ± *SMARCA4* restoration measured by GC/MS. Each line denotes a metabolite. **b** Heatmap showing relative cell viability of indicated cell lines after culturing in medium containing different concentrations of glucose or glutamine for 4 days. **c** Colony-formation assay of the indicated cell lines cultured in the presence of different concentrations of glutamine or glucose for 10–14 days. For each cell line, all dishes were fixed simultaneously. **d**, **e** Seahorse Mito Stress Test measuring mitochondrial oxygen consumption rate (OCR) in indicated cell lines cultured in the absence of glucose or glutamine. Different effects of glucose or glutamine deprivation on mitochondrial respiration. Basal respiration, maximum respiratory capacity, proton leak, and ATP production were computed in OVCAR4, H1437 (**d**), BIN-67, and H1703 (**e**) cells cultured with or without glucose or glutamine (OVCAR4, *n*  =  5; H1437, Ctrl, *n* = 6, -Glutamine, *n* = 5, -Glucose, *n* = 5; BIN-67, Ctrl, *n* = 6, -Glutamine, *n* = 6, -Glucose, *n* = 5; H1703, *n*  =  6 independent experiments). One-way ANOVA corrected for multiple comparisons. *p* values: OVCAR4, -Glutamine basal, 0.033, -Glutamine ATP - 0.0009; H1437, -Glutamine basal, 0.0154; others <0.0001. **f** Colony-formation assay of H1703 cells ± *SMARCA4* restoration cultured in medium containing different concentrations of glucose or glutamine for 10-15 days. **g** Differential abundance of metabolites measured by GC/MS in BIN-67 cells cultured in the absence of glucose or glutamine. TCA intermediates are indicated in solid gray circles. **h** Steady-state abundance of metabolites measured by GC/MS in BIN-67 cells ± *SMARCA4* restoration (two-tailed t-test, *n* = 4 indepe*n*dent experiments). *p* values (left to right): 0.0458, 0.0005, 0.0335, 0.0051, 0.0583, 0.0051, 0.0475, 0.0619, 0.0143, 0.0074, 0.0094, 0.0153, 0.501). A4: SMARCA4; A4/2: SMARCA4/2; Pro: Proficient; Def: Deficient. *p < 0.05, **p < 0.01, ***p < 0.001, ****p < 0.0001. Error bars, mean ± SD.

In addition to glucose, cancer cells can utilize other carbon sources to fuel the tricarboxylic acid (TCA) cycle sustaining biosynthesis and energy production^50,51^. To understand how SMARCA4/2-deficient cells adapt to suppressed glycolysis, we profiled steady-state abundances of 70 key metabolites related to the TCA cycle in BIN-67 cells ± SMARCA4 restoration using gas chromatography/mass spectrometry (GC/MS). In line with our above findings, glycolysis metabolites (glucose, lactate, 2- phosphoglyceric acid, 3-phosphoglyceric acid) were lower in SMARCA4/2-deficient cells compared to SMARCA4-restored cells, whereas glutamine and glutamate, amino acids that can sustain the TCA cycle once metabolized to α-ketoglutarate (α-KG)^52^, were more abundant in SMARCA4/2-deficient cells (Fig. 3a), suggesting that SMARCA4/2-deficient cancer cells are capable of switching to glutamine as their key fuel. Consistent with this interpretation, glutamine deprivation impaired cell viability, growth, and OXPHOS of SMARCA4/2-deficient ovarian and NSCLC cells much more potently than glucose depletion, while this was reversed in proficient controls (Fig. 3b–e, Supplementary Fig. 3a, b); glutamine deprivation also selectively induced strong apoptosis in SMARCA4/2-deficient cells (Supplementary Fig. 3c, d). Re-expression of SMARCA4 or SMARCA2 in SMARCA4/2-deficient cells conferred resistance to glutamine deprivation-induced apoptosis, growth suppression and OXPHOS inhibition but increased sensitivity to glucose deprivation (Supplementary Fig. 3e–k, Fig. 3f). Furthermore, steady-state abundances of all TCA cycle intermediates were more severely reduced in BIN-67 cells deprived of glutamine than those deprived of glucose (Fig. 3g, h, Supplementary Fig. 4a, b); SMARCA4-restoration in BIN-67 cells strongly elevated steady-state glucose levels accompanied with increased glycolytic intermediates (lactate, pyruvate) and reduced glutamine and glutamate levels (Fig. 3h, Supplementary Fig. 4c, d). These results suggest that SMARCA4/2 loss shifts cancer cells to utilize glutamine as a carbon source for energy supply.

**Fig. 4:**
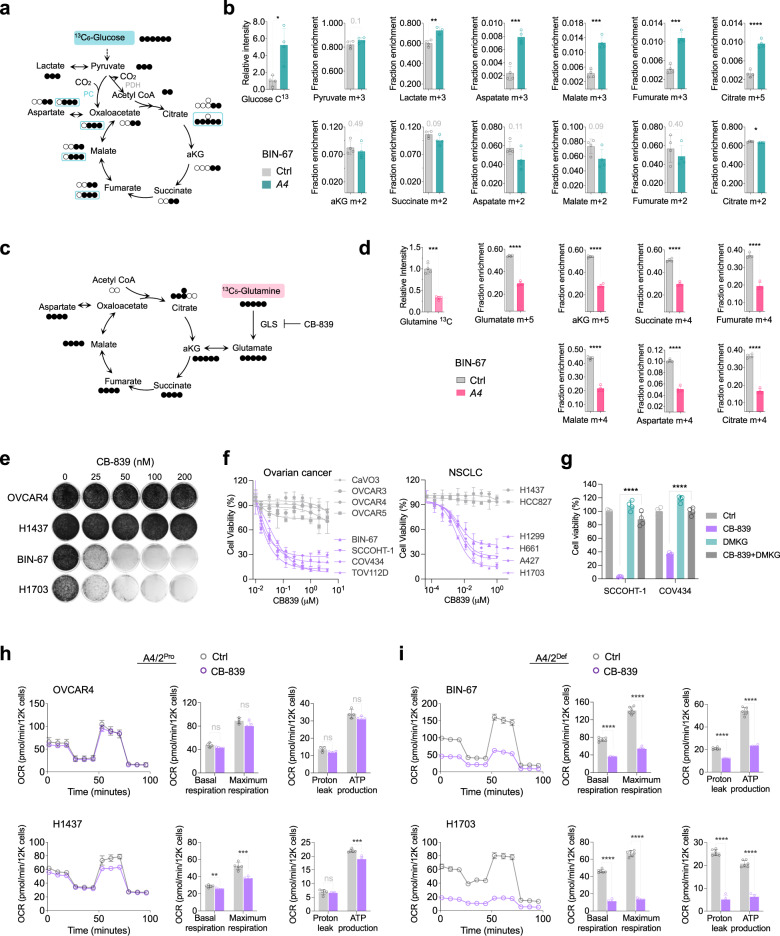
*SMARCA4/2*-loss drives the metabolic shift preferring glutamine than glucose to sustain the TCA cycle. **a** Isotope tracing diagram for ^13^C_6_-glucose through glycolysis and into the TCA cycle via pyruvate carboxylase (PC, cyan) and pyruvate dehydrogenase (PDH, grey) pathways. Solid circle, ^13^C; open circle, ^12^C. **b** Relative total labeled ^13^C_6_-glucose and fractional isotopic incorporation of ^13^C_6_-glucose in BIN-67 cells ± *SMARCA4* restoration (two-tailed t-test, *n*  =  4 independent experiments). *p* values (top): 0.0198, 0.1007, 0.0073, 0.0003, 0.0003, 0.0002, <0.0001; *p* values (bottom): 0.4937, 0.0879, 0.1047, 0.0893, 0.4046, 0.0489. **c** Diagram of stable isotope tracer analysis using uniformly labeled ^13^C_5_-glutamine. Solid circle, ^13^C; open circle, ^12^C. **d** Fractional isotopic incorporation of ^13^C_5_-glutamine into TCA cycle intermediates were measured by GC/MS (two-tailed t-test, *n*  =  4 indepe*n*dent experiments, *p* values: glutamine, 0.0007; others <0.0001) in BIN-67 cells ± *SMARCA4* restoration, cultured in ^13^C_5_-glutamine containing medium for 1 h. m, number of labeled carbons. *n*  =  4 independent experiments. **e** Colony-formation assay of indicated cell lines ± glutaminase inhibitor (CB-839) for 10-15 days. **f** Cell viability of a panel of ovarian (3 days, *n*  =  4 independent experiments) and lung (7 days, *n*  =  3 independent experiments) cancer cell lines cultured in the presence of different concentrations of CB-839. **g** Cell viability of indicated cell lines cultured with or without CB-839 (0.5μM) or DMKG (2 mM), a cell permeable analog of α-KG, for 3 days (*n*  =  4 independent experiments). Two-tailed t-test, all *p* values < 0.0001. **h**, **i** Seahorse assay measuring mitochondrial OCR in indicated cell lines in the presence of CB-839 (100 nM) for 24 h. Basal respiration, maximum respiratory capacity, proton leak, and ATP production were computed to quantify effects of CB-839 on mitochondrial respiration (OVCAR4, Ctrl, *n* = 4, CB-839, *n* = 5; H1437, Ctrl, *n* = 5; H1703, *n*  =  6; BIN-67, *n*  =  6; H1703, Ctrl, *n* = 6, CB-839, *n* = 5 independent experiments). Two-tailed t-test. *p* values: H1437, basal, 0.0029, maximum, 0.0006, ATP, 0.0002; others <0.0001. A4: SMARCA4; A4/2: SMARCA4/2; Pro: Proficient; Def: Deficient. *p < 0.05, **p < 0.01, ***p < 0.001, ****p < 0.0001. ns, not significant. Error bars, mean ± SD.

To further support that SMARCA4/2 loss induces the metabolic shift from consuming glucose to glutamine, we performed stable isotope tracer analysis (SITA) using ^13^C_6_-glucose and ^13^C_5_-glutamine. SITA using ^13^C_6_-glucose confirmed that glucose uptake was increased in BIN-67 cells upon SMARCA4-restoration, leading to overall increased metabolic flux through glucose (lactate) and TCA cycle intermediates (citrate, fumarate, malate, aspartate) via pyruvate carboxylation (Fig. 4a, b, m + 3 metabolites). Similar overall results were also obtained in H1703 cells with the restoration of SMARCA4 or SMARCA2 while each showed a unique preference for some metabolites (Supplementary Fig. 4e, f). Conversely, the ^13^C_5_-glutamine SITA in BIN-67 cells revealed that SMARCA4-restoration decreased glutamine uptake and utilization via glutaminolysis to fuel the TCA cycle as indicated by reduced glutamine metabolites (glutamate, α-KG, m + 5 metabolites) and TCA cycle intermediates (succinate, fumarate, malate, aspartate, m + 4 metabolites) (Fig. 4c, d). These observations were also confirmed in H1703 cells with ectopic expression of SMARCA4 and/or SMARCA2 (Supplementary Fig. 4g, h). Together, these data support that SMARCA4/2-loss induces a metabolic reprogram shifting from glucose to glutamine as a main fuel to sustain the TCA cycle and energy supply.

### SMARCA4/2-deficient cancer cells are highly sensitive to glutaminase inhibition

Given the critical role of glutaminolysis for sustaining the TCA cycle in SMARCA4/2-deficient cancer cells, we hypothesized that they are sensitive to the inhibition of glutaminase, which catalyzes the conversion of glutamine to glutamate (Fig. 4c), the first step in glutamine metabolism^53^. Indeed, treatment with a selective glutaminase inhibitor CB-839^54^ strongly suppressed cell viability and proliferation (Fig. 4e–g, Supplementary Fig. 5a, b), reduced OXPHOS (Fig. 4h, i), and induced apoptosis (Supplementary Fig. 5c) in SMARCA4/2-deficient ovarian and lung cancer cell lines, but not in proficient controls. This growth suppressive effect of CB-839 in SMARCA4/2-deficient cells was prevented by addition of a cell permeable analog of α-KG (Fig. 4g), supporting the critical contribution of glutamine metabolism in fueling the TCA cycle. Furthermore, re-expression of SMARCA4 or SMARCA2 conferred resistance to CB-839 (Supplementary Fig. 5d–i). These results are in line with above glutamine deprivation data, supporting that glutamine dependency in SMARCA4/2-deficient cancer cells can be exploited therapeutically.

### SMARCA4/2-deficient cancer cells rely on elevated expression of glutamine transporter SLC38A2

**Fig. 5:**
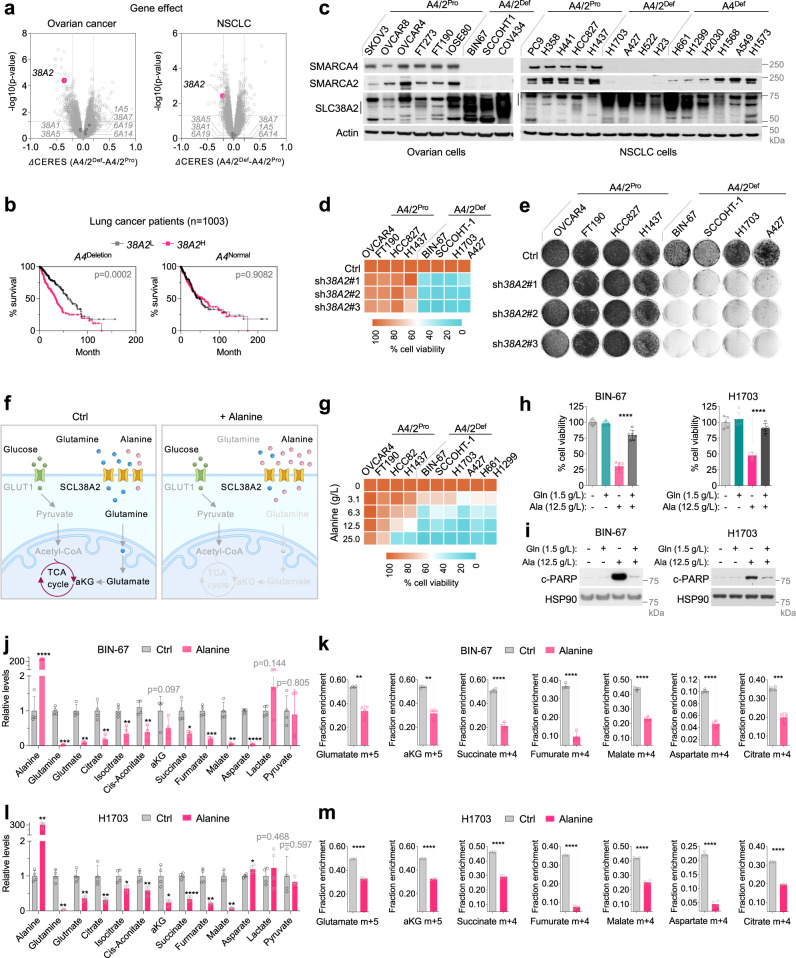
*SMARCA4/2*-deficient cancer cells rely on elevated SLC38A2 to import glutamine which can be targeted by alanine through competition. **a** Volcano plots showing the differential gene dependency between *SMARCA4/2*-deficient (*A4/2*^Def^) and proficient (*A4/2*^Pro^) ovarian (left) and lung (right) cancer cell lines, using CERES gene effect data from DepMap CRISPR-knockout screens. Two-tailed t-test. Each dot represents a gene. *SLC38A2* (*38A2*). **b** Survival of lung cancer patients (*SMARCA4*^Deletion^, *n* = 456; *SMARCA4*^Normal^, n = 547) stratified by *SLC38A2* expression (log-rank test). **c** Cell line immunoblots. The vertical lines indicate SCL38A2 range. **d** Heatmap showing cell viability of indicated cell lines expressing vector control or shRNAs targeting *SLC38A2* (7 days). **e** Colony-formation assay of indicated cell lines expressing vector control or shRNAs targeting *SLC38A2* (10–15 days). **f** A scheme depicting that alanine supplementation targets glutamine dependency of *SMARCA4/2*-deficient cancer cells. **g** Heatmap showing viability of cell lines cultured with different doses of alanine for 7 days. **h**, **i** Cell viability (**h**) and immunoblot (**i**) of BIN-67 and H1703 cells (*n*  =  5 independent experiments) cultured with indicated doses of alanine or glutamine for 4 (**h**, two-tailed t-test, *p* < 0.0001) or 3 (**i**) days. **j**, **l** Relative abundance of metabolites measured by GC/MS in BIN-67 (**j**) and H1703 (l) cells after treatment with alanine (6.25 g/L) for 14 h. **k**, **m** Fractional isotopic incorporation of ^13^C_5_-glutamine into TCA intermediates measured by GC/MS. BIN-67 (**k**) and H1703 (**m**) cells were pretreated with alanine (6.25 g/L) for 14 h and then cultured in medium containing ^13^C_5_-glutamine and alanine (6.25 g/L) for 1 h. m, number of labeled carbons. **j**–**m**: two-tailed t-test, *n*  =  4 indepe*n*dent experiments. *p* values for j, l: BIN-67, from left to right, <0.0001, 0.0004, 0.0017, 0.0038, 0.0024, 0.0022, 0.0978, 0.0146, 0.0003, 0.0043, <0.0001, 0.1435, 0.8053; H1703, from left to right, 0.0033, 0.0015, 0.0022, 0.0164, 0.0224, 0.0094, 0.0102, <0.0001, 0.0035, 0.0011, 0.0367, 0.4681, 0.5966. *p* values for **k**, **m**: BIN-67, glutamine, 0.0032, aKG, 0.0015, citrate, 0.0003, other <0.0001; H1703, all <0.0001). *p < 0.05, **p < 0.01, ***p < 0.001, ****p < 0.0001. Error bars, mean ± SD.

Our data indicate that SMARCA4/2-deficient cancer cells rely on glutamine uptake as an alternative carbon source fueling the TCA cycle. To identify glutamine transporters critical for these cancer cells, we analyzed the above DepMap CRISPR screen datasets (Fig. 1a) and identified *SLC38A2*, but not the other 7 amino acid transporters known to transport glutamine^55–57^, among the highly ranked candidates whose knockout was selectively lethal to SMARCA4/2-deficient ovarian cells (Fig. 5a, left). Similar results were obtained from DepMap datasets across 74 NSCLC cell lines stratified based on *SMARCA4* mutation status and *SMARCA2* mRNA expression (Fig. 5a, right). Notably, higher *SLC38A2* expression is associated with poor survival in lung cancer patients whose tumors exhibited *SMARCA4* deletion, but not in patients whose tumors did not harbor *SMARCA4* alterations (Fig. 5b). These observations suggest that SMARCA4/2-deficient cancer cells depend on SLC38A2 for their survival.

Consistent with its potential dominant role, *SLC38A2* was the most highly expressed glutamine transporter that was also elevated in SMARCA4/2-deficient cells compared to controls in cell lines used in above DepMap analyzes (Supplementary Fig. 6a, b). This association was also observed at the protein levels in our cell line panels (Fig. 5c). Furthermore, *SLC38A2* was the only glutamine transporter significantly elevated in SCCOHT patient tumors compared to HGSOCs (Supplementary Fig. 6c); this was also observed in NSCLC tumors expressing lower levels of *SMARCA4/2* (Supplementary Fig. 6d, e). However, restoration of SMARCA4 or SMARCA2 in SMARCA4/2-deficient cells did not consistently suppress SLC38A2 expression (Supplementary Fig. 6f, g), suggesting that the elevated SLC38A2 levels in SMARCA4/2-deficient cancer cells may be an adaptation to suppressed glycolysis rather than a direct transcriptional regulation. This likely occurred during tumor development, potentially through the selection of cells naturally expressing high levels of SLC38A2 or adaptive metabolic reprogramming that remain to be investigated.

Validating this genetic dependency of SMARCA4/2-loss, the knockdown of SLC38A2 using multiple independent shRNAs strongly reduced cell viability and proliferation (Fig. 5d, e, Supplementary Fig. 7a-c). This growth suppressive effect of SCL38A2 knockdown was rescued by ectopic expression of a RNAi-resistant *SLC38A2* cDNA (Supplementary Fig. 7d, e). Furthermore, suppression of SCL38A2 also induced apoptosis (Supplementary Fig. 7f) and suppressed OXPHOS (Supplementary Fig. 7g–k) in SMARCA4/2-deficient cancer cells but not in controls, phenocopying glutamine deprivation. While SLC38A2 also mediates alanine uptake^57,58^, alanine deprivation did not impact the growth of SMARCA4/2-deficient cancer cells (Supplementary Fig. 7l, m), supporting the dominant contribution of glutamine in this context. Together, these data show that SMARCA4/2-deficient cancer cells depend on elevated SLC38A2 for glutamine uptake and metabolism to sustain the TCA cycle.

### Alanine restricts SLC38A2-dependent glutamine import and metabolism through competition

While above data validate SLC38A2 as a synthetic lethal target of SMARCA4/2-loss, no SLC38A2 inhibitor is currently available. Given that alanine is also imported by SLC38A2^57,58^, we explored the potential of suppressing glutamine uptake through competition with alanine (Fig. 5f). Alanine supplementation induced dosage-dependent growth suppression in SMARCA4/2-deficient cancer cells but had little or mild effect in proficient controls (Fig. 5g, Supplementary Fig. 8a–c). Mirroring the effects of inhibitors targeting Complex I and glutaminase or glutamine deprivation, alanine supplementation selectively suppressed OXPHOS in SMARCA4/2-deficient cancer cells (Supplementary Fig. 8d–g). Supporting that alanine supplementation competes with glutamine for SLC38A2, the addition of glutamine or ectopic expression of SLC38A2 rescued SMARCA4/2-deficient cancer cells from growth suppression and OXPHOS inhibition caused by alanine treatment (Fig. 5h, Supplementary Fig. 8d–n). Like glutamine deprivation, alanine supplementation also induced strong apoptosis in SMARCA4/2-deficient cells (Supplementary Fig. 8c), which was prevented by the additional supply of glutamine or ectopic expression of SLC38A2 (Fig. 5i, Supplementary Fig. 8o, p). These data suggest that alanine suppresses the growth of SMARCA4/2-deficient cancer cells by restricting SLC38A2-dependent glutamine import.

Supporting this, alanine supplementation significantly reduced steady-state levels of intracellular glutamine, glutamate, and TCA cycle intermediates without impacting glycolysis metabolites in BIN-67 cells (Fig. 5j, Supplementary Fig. 8q–s). Furthermore, ^13^C_5_-glutamine SITA confirmed that glutamine uptake was markedly suppressed in BIN-67 cells cultured with alanine supplementation (Supplementary Fig. 8t), accompanied with decreased glutaminolysis fueling TCA cycle as indicated by significantly reduced m + 5 glutamine metabolites (glutamate, a-KG) and m + 4 TCA cycle intermediates (Fig. 5k). Similar results were also obtained in H1703 cells (Supplementary Fig. 8u, Fig. 5l, m). Together, these data demonstrate that alanine could be used to target glutamine dependency in SMARCA4/2-deficient cancer cells by suppressing SLC38A2-mediated glutamine import and consequently, inhibiting glutaminolysis and OXPHOS.

### Inhibitors targeting OXPHOS or glutaminase and alanine supplementation-based treatments suppress SMARCA4/2-deficient tumors

**Fig. 6:**
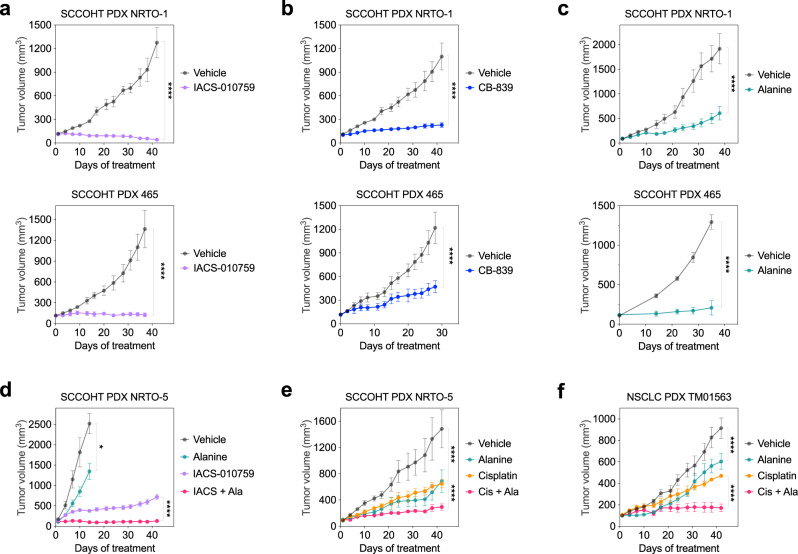
Inhibitors targeting OXPHOS or glutaminase, and alanine supplementation-based treatments suppress the growth of SMARCA4/2-deficient tumors. **a**–**c** Tumor volumes of mice bearing tumors of SCCOHT PDX models (top, NRTO-1; bottom, 465) treated with IACS-010759 (**a** 7.5 mg/kg), CB839 (**b** 200 mg/kg), alanine (**c** 4 g/kg), or their matching vehicle controls (*n* = 5–8 for each arm, except for PDX 465, alanine *n* = 4). **d** Tumor volumes of mice bearing SCCOHT PDX NRTO-5 tumors treated with vehicle, IACS-010759 (7.5 mg/kg), alanine (4 g/kg) or their combination (*n* = 4 for each arm). **e**, **f** Tumor volumes of mice bearing SCCOHT PDX NRTO-5 (**e**) or SMARCA4/2-deficient NSCLC PDX TM01563 (**f**) tumors treated with vehicle, cisplatin (2 mg/kg), alanine (4 g/kg), or their combination (*n* = 4 for each arm). *p* values: all <0.0001 except for alanine group vs vehicle in (**d**), which is 0.0113. IACS, IACS-010759; Ala, alanine; Cis, cisplatin. Two-way ANOVA, *p < 0.05, ****p < 0.0001. Error bars, mean ± SEM.

Our study has revealed multiple druggable vulnerabilities of SMARCA4/2-deficient cancer cells, exploiting their metabolic shift from glucose to glutamine as a key carbon source. To validate in vivo, we first evaluated the anti-tumor effects of IACS-010759 and CB-839 in mouse xenograft models of SCCOHT. As single agents, both inhibitors strongly suppressed tumor growth in multiple cell line xenografts (Supplementary Fig. 9a–d) and patient-derived xenograft (PDX) models (Supplementary Fig. 9e, Fig. 6a, b), suggesting that IACS-010759 and CB-839, both currently being evaluated in clinical trials, are potential treatment options for SCCOHT patients.

Next, we investigated the potential utility of alanine supplementation in treating SMARCA4/2-deficient tumors. Consistent with our in vitro observations, alanine supplementation alone was capable of suppressing tumor growth of two independent SCCOHT PDX models (Fig. 6c). The anti-tumor effect of alanine alone was significant but less pronounced in the third and more aggressive SCCOHT PDX model, which also responded less to IACS-010759; however, the combination of alanine and IACS-010759 strongly suppressed tumor growth (Fig. 6d) without inducing noticeable toxicity to the animals such as body weight loss (Supplementary Fig. 9f). Similar results were also obtained in a SMARCA4/2-deficient NSCLC PDX model (Supplementary Fig. 9g–i). Since conventional chemotherapy remains the first-line treatment for SMARCA4/2-deficient cancers, we evaluated the efficacy of alanine supplementation in combination with cisplatin. This combination synergistically inhibited viability and induced apoptosis in these cancer cells (Supplementary Fig. 9j, k) and strongly suppressed tumor growth of SCCOHT and NSCLC PDXs more than either agent alone (Fig. 6e, f). Taken together, our data support the use of alanine supplementation as an effective therapeutic intervention for SMARCA4/2-deficient cancers.

## Discussion

Here we uncover a metabolic shift induced by SMARCA4/2-dual loss in ovarian and lung cancers that can be exploited therapeutically. While SWI/SNF complexes are known to regulate nutrient sensing and energy metabolism during normal development^59^, their critical roles in cancer metabolism have only recently emerged. *SMARCA4*-mutant lung cancer cells with intact SMARCA2 depend on elevated OXPHOS activity by upregulating the master transcription factor PGC-1a^38^, whereas *ARID1A* inactivation in ovarian cancer cells causes reliance on glutamine metabolism through activating glutaminase expression^60^. Our study reveals that concomitant loss of SMARCA4/2, but not SMARCA4 inactivation alone, strongly downregulates GLUT1 expression resulting in repressed glucose uptake and glycolysis; consequently, SMARCA4/2-deficient cancer cells elevate SLC38A2, likely acquired during tumor development, to increase import of glutamine as an alternative oxidative fuel, leading to their addiction to glutamine metabolism and OXPHOS. While additional cell context-specific alterations induced by SMARCA4/2-loss may also contribute to the metabolic reprograming, our results support the pivotal role of GLUT1/SLC38A2 underlying the glutamine and OXPHOS dependency of these cancer cells.

Our study also establishes that this metabolic dependency of SMARCA4/2-loss can be targeted at multiple essential nodes. The Complex I inhibitor IACS-010759 and the glutaminase inhibitor CB-839 both showed strong anti-tumor activities across cell lines and PDX models with SMARCA4/2-loss, suggesting that these clinical agents^61,62^ can be effective in treating SMARCA4/2-deficicent cancers. Moreover, we demonstrate that alanine, a natural and non-toxic amino acid, can be used to compete with glutamine for SLC38A2-mediated uptake leading to suppression of glutamine metabolism and OXPHOS in these cancer cells. Alanine supplementation alone was sufficient to suppress tumor growth and elicited superior anti-tumor activities in combination with either IACS-010759 or cisplatin. A recent report of a Phase I trial evaluating IACS-010759 in patients with relapsed acute myeloid leukemia and advanced solid tumors showed a narrow therapeutic index of IACS-010759 due to dose-limiting toxicities^63^. Our findings suggest that SMARCA4/2-deficicency may serve as a biomarker for guiding patient selection for applying IACS-010759 and the addition of alanine dietary supplementation may reduce IACS-010759 dose needed to suppress tumors and minimize the toxicities, potentially enhancing its clinical utility.

The alanine dosage used here, estimated as the equivalent of 16 g/day for a 50 kg person^64^, is achievable in patients as the recommended alanine supplementation for diabetes management is 20–40 g/day^65^. While dietary restrictions such as glutamine deprivation have shown promising activities in pre-clinical cancer models, sufficiently reduced levels of targeted nutrients are generally difficult to achieve in patients^66^. In contrast, alanine, readily available at low cost, can be easily added as a dietary supplement to enhance current standard treatments for SMARCA4/2-deficent cancers. It has been shown that pancreatic cancer cells upregulate SLC38A2 to increase alanine uptake to support their metabolism by replacing glucose and glutamine to fuel the TCA cycle^25,67^. Therefore, the long-term effects of alanine supplementation require further investigation for patients with SMARCA4/2-deficient cancers along with the development of SLC38A2 inhibitors, that may also prove efficacious for other SLC38A2-dependent tumors such as pancreatic cancer^25^.

In summary, our study reveals druggable vulnerabilities of a GLUT1/SLC38A2-mediated metabolic shift due to SMARCA4/2-loss. In addition to available candidate drugs targeting Complex I and glutaminase, we develop the concept and provide proof of principle data supporting the potential clinical utility of alanine dietary supplementation for patients affected by these hard-to-treat cancers.

## Methods

All research in this study complies with all relevant ethical regulations. All biohazard protocols were approved by the Environmental Health and Safety of McGill University and the Biosafety Committee of the University of British Columbia (UBC). All animal procedures were approved by the Facility Animal Care Committee (FACC) of McGill University and the Animal Care Committee of UBC, according to guidelines of the Canadian Council on Animal Care Standards (CCAC). Studies on SCCOHT patient tumors were approved by the Institutional Review Board (IRB) at McGill University, McGill IRB # A08-M61-09B, and UBC, IRB# H18-01652.

### Data mining and analysis of genome-wide CRISPR screen data

CRISPR/Cas9 knockout screening data were downloaded from the DepMap Public 21Q2 dataset (https://depmap.org/portal/). The genetic background and *SMARCA4/2* expression of DepMap cell lines were derived from Cancer Cell Line Encyclopedia. Cell lines were called as *SMARCA4/2*-dual deficient based on literature references (BIN-67, SCCOHT-1, COV434, TOV112D, OVK18, H1703, A427, H23, H522)^13,29,36,68^ or if the cell lines exhibited low gene expression (Log2(TPM + 1) < 3) of *SMARCA2* and exhibited damaging mutations on *SMARCA4* (see Supplementary Data 1). Differential dependency was calculated by comparing CERES scores between *SMARCA4/2*-dual deficient cell lines versus proficient lines. Cell lines with *SLC38A2* mutation were excluded. Significance was assessed using unpaired two-tailed t-test.

### Cell culture

293 T cells were cultured with DMEM (Dulbecco’s modified Eagle medium, Thermo Fisher Scientific, Cat# 11995-065) containing 7% fetal bovine serum (Sigma, Cat# F1051), 1% penicillin/streptomycin (Thermo Fisher Scientific, Cat# 15140-122), and 2 mM L-glutamine (Thermo Fisher Scientific, Cat# 25030-081). All other cell lines were cultured in RPMI (Roswell Park Memorial Institute 1640 Medium, Thermo Fisher Scientific, Cat# 11875-093; no pyruvate) with 7% fetal bovine serum (Sigma, Cat# F1051), 1% penicillin/streptomycin (Thermo Fisher Scientific, Cat# 15140-122), and 2 mM L-glutamine (Thermo Fisher Scientific, Cat# 25030-081). Cells were maintained at 37 °C in a humidified 5% CO_2_-containing incubator and a regular Mycoplasma test was performed using Mycoalert Detection Kit (Lonza, Cat # LT07-318). OVCAR4: Dr. E. Wang (University of Calgary, Calgary, originally from NCI); BIN-67: Dr. B. Vanderhyden (Ottawa Hospital Research Institute, Ottawa) originally from Dr. S.R. Goldring (Hospital for Special Surgery, New York, originally derived from patients with ovarian carcinoma treated at the Dana-Farber Cancer Institute (Boston, MA)); SCCOHT-1: Dr. R. Hass (Medical University Hannover, Hannover, generated by Dr. R. Hass); PC9: Dr. R. Bernards (Netherlands Cancer Institute, Amsterdam, originally from Immuno-Biological Laboratories (IBL), Tokyo, Japan); OVCAR8, SKOV3: Dr. M. Witcher (McGill University, Montreal, originally from ATCC); IOSE80: Dr. N. Auersperg (The University of British Columbia, Vancouver); FT237, FT190: Dr. T.G. Shepherd (The Mary & John Knight Translational Ovarian Cancer Research Unit, Ontario, FT190 was originally provided by R. Drapkin, University of Pennsylvania, Philadelphia, PA); 293 T: ATCC, CRL-3216; H1703: ATCC, CRL-5889; H1299: ATCC, CRL-5803; H1437: ATCC, CRL-5872; HCC827: ATCC, CRL-2868; A549: ATCC, CCL-185; H2030: ATCC,CRL-5914; H1568: ATCC,CRL-5876; H661: ATCC,HTB-183; H23: ATCC, CRL-5800; A427: ATCC, HTB-53; H522: ATCC, CRL-5810; H358: ATCC, CRL-5807; H441: ATCC, HTB-174; H1792: ATCC, CRL-5895; H1573: ATCC, CRL-5877; COV434: Sigma, 07071909; TOV-112D: Anne-Marie Mes-Masson (Centre de recherche CHUM et; Institut du cancer de Montréal); CaOV3: Dr. Nelly Auersperg (University of British Columbia, Vancouver, originally from Jorgen Fogh, MSKCC); OVCAR3, OVCAR4 and OVCAR5: Dr. Nelly Auersperg (University of British Columbia, Vancouver, originally from Thomas C Hamilton, Fox Chase Cancer Centre). All cell lines have been validated by Short-Tandem Repeat profiling.

### Compounds and antibodies

IACS-010759 (S8731), CB839 (S7655), oligomycin A (S1478), rotenone (S2348), BAY-876 (S8452), and cisplatin (S1166) were purchased from Selleck Chemicals (Houston, Texas, USA). Alanine (550-001-EG) was obtained from Wisent Bio (Montreal, QC, Canada). ^13^C_5_-Glutamine (CLM-1822), ^13^C_6_-Glucose (CLM-1396), were from Cambridge Isotopes Laboratories (Tewksbury, MA). Metformin (#13118) and Phenformin (#14997) were from Cayman Chemical (Ann Arbor, MI, USA). Dimethyl-α-ketoglutarate (DMKG, #349631) was from Sigma (Oakville, ON, Canada). Caspase3/7 green dye (4440) was from Sartorius (Goettingen, Germany). Antibodies against HSP90 (H-114, 1:1,0000) and β-Actin (Cat# sc-47778, 1:1,0000) were from Santa Cruz Biotechnology (Dallas, TX, USA); antibodies against cleaved PARP (Cat# 5625, 1:1,000) and SMARCA2 (Cat# 11996, 1:1,000) were from Cell Signaling (Danvers, MA, USA); antibody against SMARCA4 (A300-813A, 1:1,000) and (ab110641, 1:5000) were from Bethyl Laboratories (Montgomery, TX, USA) and Abcam (Tornoto, ON, Canada), respectively; antibody against SLC2A1 (ab15309, 1:1,000) was from Abcam; antibody against SLC38A2 (BMP081, 1:1,000) was from MBL (Woburn, MA, USA). Antibodies for immunohistochemistry are listed in the corresponding method section below.

### Plasmids, Lentivirus production and infection

Individual shRNA vectors used were from the Mission TRC library (Sigma) provided by McGill Platform for Cellular Perturbation (MPCP) at McGill University: sh*SMARCA2*#1 (TRCN0000358828); sh*SMARCA2*#2 (TRCN0000020333); sh*SLC38A2*#1 (TRCN0000020241), sh*SLC38A2*#2 (TRCN0000020242), sh*SLC38A2*#3 (TRCN0000020243). sgRNA (ATGGTGCTGACCCCCAGGCCTT) targeting *SMARCA4* was cloned into pLentiCRISPRv2 which was from Addgene (Cat# 52961). The GLUT1-eGFP plasmid was a gift from Wolf Frommer (Addgene plasmid #18729). The pLX304-*GFP* and pLX304-*SLC38A2* were obtained from TRC3 ORF collections from TransOMIC and Sigma provided by MPCP. pReceiver control vector, pReceiver-*SMARCA2*, and pReceiver-*SMARCA4* were purchased from GeneCopoeia. pIN20 and pIN20-*SMARCA4* were kindly provided by Dr. Jannik N. Andersen (The University of Texas, MD Anderson Cancer Center).

Lentiviral transduction was performed using the protocol as described at http://www.broadinstitute.org/rnai/public/resources/protocols. Briefly, 2.5 × 10^6^ 293 T cells were seeded in six-well plate with 2 mL DMEM medium per well. 8 hours later, cells were transfected with indicated lentiviral constructs, the packaging (psPAX2) and envelope (pMD2.G) plasmid by CaCl_2_. Virus containing medium were collected (24 and 36 h after transfection) and stored at −80 °C. Infected cells (~8 h for infection and ~20 h for recovery) were selected in medium containing puromycin or blasticidin for 2–3 days and collected immediately for the experiments.

### Colony formation assays

Single-cell suspensions of all cell lines were counted and plated into 6-well plates at a density of 0.5–8 × 10^4^ cells per well. Cells were cultured in a medium containing the indicated drugs for 10–14 days (refreshed every 3 days). At the endpoints, cells were fixed with 4% formaldehyde in PBS, stained with crystal violet (0.1%w/v in water) and photographed.

### Cell viability assays

Cultured cells were plated into 96-well plates (0.5k–6k cells per well) and treated with medium containing the indicated drugs the next day. For Fig. 1a, Extended Fig. 1j, k, n, and Extended Fig. 5a, c, cells were fixed in methanol: acetic acid: water solution (1:1:8) and then stained with 0.5% crystal violet in methanol after 3 days of treatment; the absorbed dye was resolubilized with 10% acetic acid and measured spectrophotometrically at 595 nM; cell survival was calculated by normalizing the absorbance to that of DMSO-treated controls. For all the remaining cell viability assays, cells were cultured for 4–7 days (refreshed twice a week), and cell viability was measured using the CellTiter-Blue® Viability Assay (Promega) by measuring the fluorescence (560/590 nm) in a microplate reader. Relative cell viability was calculated by normalizing the absorbance or fluorescence to that of the vehicle-treated controls after background subtraction. Heatmaps for cell viability were generated with pheatmap (1.0.12).

### Protein lysate preparation and immunoblots

Cells were lysed with protein sample buffer, heated at 95 °C for 5 min, and processed with Novex® NuPAGE® Gel Electrophoresis Systems (Thermo Fisher Scientific). For GLUT1 and SLC38A2 detection, unboiled protein lysates were used. HSP90 and beta-actin served as loading controls.

### Caspase3/7 staining and IncuCyte imaging

Cells were seeded in 96-well plates (0.5–8k) and treated with different drugs. IncuCyte® live-cell analysis imaging system was used to record images every 4 h. Cell proliferation was determined by phase-contrast images based on cell confluence. For cell apoptosis, caspase-3/7 green dye (Sartorius, Cat# 4440) was added to the culture medium and apoptosis was analyzed based on fluorescent staining of apoptotic cells.

### RNA isolation and qRT-PCR

Total RNA was isolated using Trizol (Invitrogen) and converted to cDNAs using the Maxima First Strand cDNA Synthesis Kit (Thermo Scientific). Quantitative real-time reverse transcription PCR (qRT-PCR) was carried out using SYBR® Green master mix (Roche) according to manufacturer protocols. Relative mRNA levels of indicated genes were normalized to the housekeeping gene *ACTB*. The sequences of the primers used for qRT-PCR are as follows:

ACTB_Forward (Fwd), GTTGTCGACGACGAGCG;

ACTB_Reverse (Rev), GCACAGAGCCTCGCCTT;

SLC2A1_Forward (Fwd), AGGTGATCGAGGAGTTCTAC;

SLC2A1_Reverse (Rev), TCAAAGGACTTGCCCAGTTT;

SLC38A2_Forward (Fwd), TCCTGTTAAGTGGTGTACTGGT;

SLC38A2_Reverse (Rev), CCAGGTGCATTGTGTACCCA;

### Transcriptome analysis

Cell lines: RNA-seq data of cell lines with genetic perturbation of SMARCA4/2 used in this study are: BIN-67 cells ± restoration of SMARCA4 or SMARCA2 (GSE117735^42^,), A427 cells ± SMARCA4 restoration (GSE151026^43^,), H1944 cells ± SMARCA2 knockdown (GSE144843^44^,). Sequencing files were downloaded from Sequence Read Archive (SRA) and mapped to the reference human genome sequence (hg38) with STAR (2.6.1c)^69^. Gene expressions were calculated by Homer^70^ with Gencode gene annotation GTF file. Differential expression genes were identified with DESeq2 (version 1.19.38)^71^. The transcriptome data of DepMap cancer cell lines were derived from DepMap and DESeq2 was applied to determine the differential expression genes.

Patient tumors: RNA-seq data of 13 SCCOHT and 21 HGSOC patient tumors were obtained as described previously^18,26^. RNA-seq read counts of 379 ovarian cancer tumors were obtained from UCSC xena (http://xena.ucsc.edu/). RNA-seq data of 1016 lung cancer tumors were obtained from cBioportal. Sequencing results were processed by following mRNA quantification analysis pipeline of Genomic Data Commons (https://docs.gdc.cancer.gov/Data/Bioinformatics_Pipelines/Expression_mRNA_Pipeline/): first aligning reads to the GRCh38 reference genome with STAR-2.6.0c and then by quantifying the mapped reads with HTSeq-0.6.1^72^.

### Mitochondrial respiration and glycolysis measurements

OCR and ECAR experiments were performed using the XFe96 or XFe24 Analyzer apparatus from Seahorse Bioscience. Cells were cultured in 6 well plate, treated with the indicated drugs or vehicle for indicated time. Then treated cells are reseeded (5000 to 40,000 cells/well) on Seahorse XFe96 V3 PET plates (Agilent, 101085-004) or XFe24 plates (Agilent, Cat# 100882-004) the day before the experiment. The next day, the medium was exchanged with RPMI supplemented with either 2 mM glutamine and 10 mM glucose (Mito Stress Test) or 2 mM glutamine (Glycolysis Stress Test) adjusted to pH~7.4. The plates were incubated for 60 min at 37 °C in a CO_2_-free incubator before loaded on the Seahorse Analyzer. For the mitochondrial respiration test, oligomycin, FCCP, and a mixture of antimycin A and rotenone were injected to a final concentration of 2 μM, 0.5 μM, and 3 μM, respectively. For Extended Fig. 1c, e, the drugs were sequentially added at indicated time point of 20 mins, 40 mins, and 60 mins. For all the remaining mitochondrial respiration assays, the drugs were sequentially injected at indicated time point of 25 mins, 50 mins, and 75 mins. For the glycolysis stress test, glucose, oligomycin, and 2-deoxyglucose were injected to a final concentration of 10 mM, 2 μM, and 100 mM, at indicated time point of 20 mins, 40 mins and 60 mins, respectively. OCR and ECAR values of each cell line were normalized to cell numbers seeded.

### Glucose uptake assay

Glucose uptake was determined using the Glucose Uptake-Glo assay (Promega) following the manufacturer protocols. Briefly, cells were seeded in 96-well plates (5000 cells/well) the day prior to the assay. The next day, the medium was removed, washed with PBS, and incubated in 1 mM 2-deoxy-glucose for 10 min at room temperature. Cells were lysed and neutralized before the addition of the kit’s detection reagent. Luminescence was measured on a luminometer.

### Metabolite profiling and isotope tracing

Metabolites were profiled at the Rosalind and Morris Goodman Cancer Institute Metabolomics Innovation Resource. For metabolite profiling experiments, cells were plated in RPMI supplemented with 6% dialyzed FBS (Wisent Bio, Cat# 080-950) at ~80% confluency the day before experiments. For isotope metabolic tracing, media was replaced with glutamine- or glucose-free RPMI supplemented with 6% dialyzed FBS and ^13^C_5_-glutamine or ^13^C_6_-glucose (Cambridge Isotopes Laboratories, Tewksbury, MA) for 1 hr or 0.5 hr, respectively. In addition, dishes were kept in unlabeled media as control. Cells were washed twice in cold saline solution (NaCl, 0.9 g/l) and metabolites were extracted with 1 ml 80% ice-cold methanol (GC/MS grade). After 2 rounds 10 min sets of sonication (30 seconds on/30 seconds off at high intensity) on slurry ice using a Bioruptor UCD-200 sonicator, the homogenates were centrifuged at 14,000 × *g* at 4 °C for 10 min. Supernatants were collected and supplemented with internal control (800 ng myristic acid-D27) and dried in a cold vacuum centrifuge (Labconco) overnight. The dried pellets were reconstituted with 30 μL of 10 mg/mL methoxyamine-HCl in pyridine, incubated for 30 min at room temperature. Samples were then derivatized with MTBSTFA for 30 min at 70 °C. A volume of 1 μL of sample was injected splitless with an inlet temperature of 280°C into the GC/MS instrument (5975 C, Agilent). Metabolites were resolved by separation on DB-5MS + DG (30 m x 250 µm x 0.25 µm) capillary column (Agilent J&W, Santa Clara, CA, USA). Helium was used as the carrier gas with a flow rate such that myristic-d27 acid eluted at approximately 18 min. The quadrupole was set at 150˚C, the source was at 230 °C and the GC/MS interface at 320˚C. The oven program started at 60˚C held for 1 min, then increased at a rate of 10˚C/min until 320˚C. Bake-out was at 320˚C for 9 min. Metabolites were ionized by electron impact at 70 eV. All samples were injected three times: twice using scan (50–1000 m/z) mode (1x and 25x dilution for steady state samples or 1x and 24x dilution for tracer samples) and once using selected ion monitoring (SIM) mode. All of the metabolites described in this study were validated against authentic standards confirming mass spectra and retention times. Integration of ion intensities was accomplished using Mass Hunter Quant (Agilent). Generally, M-57^+.^ Ions (and isotopes) were analyzed. Mass isotopomer distribution analysis was carried out using in-house software using an in-house algorithm adapted from Nanchen et al. as previously described^73^.

### Immunohistochemistry

Tissue microarrays (TMAs) of patient tumors from McGill (52 SCCOHT cases)^26^ and UBC (24 SCCOHT and 108 epithelial ovarian carcinoma cases)^34^ were as previously described. Studies on SCCOHT patient tumors were approved by the Institutional Review Board (IRB) at McGill University, McGill IRB # A08-M61-09B, and UBC, IRB# H18-01652. Informed consent was obtained from all participants in accordance with the relevant IRB approvals. For all IHC analysis, cores with low tumor cellularity and artifacts were not included in the analysis.

SCCOHT and other ovarian cancer TMAs were cut at 4 μm thickness onto Superfrost Plus glass slides, and were processed using the Leica BOND RX Stainer. Immunohistochemical (IHC) staining was performed with antibodies to GLUT1 (1:3000, ab115730, Abcam), SMARCA4 (1:2000, ab110641, Abcam), SMARCA2 (1:200, HPA029981, Sigma) and Cyclin D1 (1:400, #2978, Cell Signaling Technology). GLUT1 expression was reviewed by a pathologist (D.G.H) and quantitated using the Aperio ImageScope software. The GLUT1 positivity was defined by the counts of pixels above the threshold to all pixel counts in the tumor area analyzed.

### Survival analysis

Survival analyzes of lung adenocarcinoma patients (*n* = 1003) were performed using data derived from cBioportal (https://www.cbioportal.org/). This dataset was analyzed by first separating patients into two groups (*SMARCA4* deletion, *SMARCA4* normal) according to *SMARCA4* copy number status. Next, patients in each group were stratified into *SLC38A2* high (top 50%) and low groups (bottom 50%), followed by log-rank test. Patients with and without *SMARCA4* alterations were analyzed separately.

### Mouse xenografts and in vivo drug efficacy studies

Animal experiments were performed according to standards outlined in the Canadian Council on Animal Care Standards (CCAC) and the Animals for Research Act, R.S.O. 1990, Chapter c. A.22, and by following internationally recognized guidelines on animal welfare. All animal procedures (Animal Use Protocol #7407 (McGill) and # A17-0146 (UBC)) were approved by the Institutional Animal Care Committee according to guidelines of the CCAC. Since all human cell line-derived xenografts and PDXs were derived from female patients, all animal experiments in this study used female mice. Housing conditions: Temperature: 16 degrees min − 24 degrees max; Humidity: 15% low - 60% high; Photoperiod: 7am–7pm light, 7pm–7am dark (McGill); 6a–6pm light, 6pm–6am dark (UBC). The animal experiments carried out at Goodman Cancer Research Institute of McGill University used 8–12-week-old in-house bred female NOD.Cg-Prkdc^scid^ Il2rg^tm1Wjl^/SzJ (NSG) mice; the animal experiments carried out at British Columbia Cancer Research Institute used 7–9 week-old in house bred female NRG (NOD.Rag1KO.IL2RγcKO) mice. To establish xenograft models, COV434 cells (2×10^6^ cells/mouse) or SCCOHT PDX465 (^34^, 50 mg/mouse, homogenized and resuspended in HBSS) were injected with a 1:1 mix of matrigel (Corning) in a final volume of 200 μl subcutaneously into the back of NRG mice; BIN-67 cells (10×10^6^ cells/mouse) with a 1:1 mix of matrigel (Corning) in a final volume of 100 μl subcutaneously into the right flank of NSG mice; and SCCOHT PDX NRTO-1 and NRTO-5, established and viably preserved at Goodman Cancer Research Institute of McGill University, and Lung PDX obtained from The Jackson Laboratory (TM01563), were cut into pieces and then inserted into a pocket in the subcutaneous space of NSG mice. Mice were randomized to treatment arms once the average tumor volume reached 100 mm^3^. IACS-010759 was resuspended in 0.5% methylcellulose administrated by gavage at 7.5 mg/kg (6-on 1-off for SCCOHT PDX#2; 5-on 2-off for the remaining PDXs). CB839 was resuspended in 25% (w/v) HPBCD in 10 mM citrate (pH2) administrated by gavage at 200 mg/kg twice daily. Alanine was resuspended in 0.9% sodium chloride solution administrated by gavage at 4 g/kg daily (3 times a day, each time 250 ul). Cisplatin (SelleckChem) was resuspended in 0.9% sodium chloride solution administrated intraperitoneally at 2 mg /kg once a week (3 weeks on 1 week off). Tumor volume and mouse weight were measured twice to thrice weekly. Tumor volume was calculated as length*(width)^2^*0.52. The persons who performed all the tumor measurements were blinded to the treatment information. Experiments involving COV434 xenograft and SCCOHT PDX#2 were carried out at BCCRI. Experiments involving BIN-67 xenograft, SCCOHT PDX#1 and #3 and lung PDX#1 experiments were carried out at the Goodman Cancer Research Institute of McGill University. The maximal tumor size permitted by our Institutional Animal Care Committees is 2500 mm^3^, which was not exceeded in our experiments.

### Statistics and reproducibility

GraphPad Prism 8 software was used to generate graphs and statistical analyzes. Statistical significance was determined by one- and two-way ANOVA, Student’s t-test, log-rank test. Methods for statistical tests, the exact value of n, and definition of error bars were indicated in figure legends, *p  <  0.05, **p  <  0.01, ***p  <  0.001, and ****p  <  0.0001. For simplicity, one-way ANOVA Brown–Forsythe and Welch tests followed by Dunnett’s test for multiple comparisons is referred as “one-way ANOVA corrected for multiple comparisons” in the figure legends. All experiments have been reproduced in at least two independent experiments unless otherwise specified in the figure legends. All immunoblots and images shown are representatives of these independent experiments.

### Reporting summary

Further information on research design is available in the Nature Portfolio Reporting Summary linked to this article.

## Supplementary information

Supplementary Information

Reporting Summary

Supplementary Data 1

Supplementary Data 2

Supplementary Data 3

Supplementary Data 4

Description of Additional Supplementary Files

Peer Review File

## Data Availability

Gene expression data are obtained from the DepMap (https://depmap.org/portal/) for cell lines and downloaded from UCSC Xena (https://xenabrowser.net/datapages/) for TCGA tumors of ovarian cancer patients. Out of 13 SCCOHT patient tumors, RNA-seq data of 10 cases were obtained from a previous study^18^ and that of the other three cases can be found using the accession number EGAS00001005448^26^. Source data for RNA-seq, ChIP-seq and ATAC-seq can be found using the accession number GSE151026^43^, GSE117735^42^, GSE121755^33^, GSE144843^44^. All Raw data of GC-MS generated in this study can be found in metabolomics workbench^74^ using the Study ID ST002578 [https://www.metabolomicsworkbench.org/data/DRCCMetadata.php?Mode=Study&StudyID=ST002578&StudyType=MS&ResultType=1], ST002586 [https://www.metabolomicsworkbench.org/data/DRCCMetadata.php?Mode=Study&StudyID=ST002586&StudyType=MS&ResultType=1], and ST002587 [https://www.metabolomicsworkbench.org/data/DRCCMetadata.php?Mode=Study&StudyID=ST002587&StudyType=MS&ResultType=1]. The Supplementary Data files can be found in FigShare with the DOI 10.6084/m9.figshare.22709206. All unique materials generated are readily available from the authors. All remaining data can be found in the Article, Supplementary and Source data files. Source data are provided with this paper.

## Source data

Source Data
