# Supplementary material for: Alanine supplementation exploits glutamine dependency induced by SMARCA4/2-loss
Source: Nat Commun. 2023 May 20;14:2894. doi: 10.1038/s41467-023-38594-3 (PMC10199906; doi:10.1038/s41467-023-38594-3)
Supplement: Supplementary file 1 — Supplementary Information [file 41467_2023_38594_MOESM1_ESM.pdf]

SUPPLEMENTARY INFORMATION

Alanine supplementation exploits glutamine dependency induced by SMARCA4/2-loss

Xianbing Zhu[1, 2]*, Zheng Fu[1, 2]*, Shary Y. Chen[3, 4]*, Dionzie Ong[3], Giulio Aceto[1, 2], Rebecca Ho[3, 4], Jutta Steinberger[1, 2], Anie Monast[1, 2], Virginie Pilon[1, 2], Eunice Li[3], Monica Ta[3], Kyle Ching[3], Bianca N. Adams[1, 2], Gian L. Negri[5], Luc Choiniere[6], Lili Fu[7], Kitty Pavlakis[8], Patrick Pirrotte[9, 10], Daina Z. Avizonis[6], Jeffrey Trent[11], Bernard E. Weissman[12, 13], Ramon I. Klein Geltink[3], Gregg B. Morin[5, 14], Morag Park[1, 2], David G. Huntsman[3, 4, 15], William D. Foulkes[16, 17, 18], Yemin Wang[3, 4]§ and Sidong Huang[1, 2]§

[1]Department of Biochemistry, McGill University, Montreal, QC, Canada.
[2]Rosalind & Morris Goodman Cancer Institute, McGill University, Montreal, QC, Canada.
[3]Department of Pathology and Laboratory Medicine, University of British Columbia, Vancouver, BC, Canada.
[4]Department of Molecular Oncology, British Columbia Cancer Research Institute, Vancouver, BC, Canada.
[5]Canada's Michael Smith Genome Science Centre, British Columbia Cancer Research Institute, Vancouver, BC, Canada.
[6]Rosalind & Morris Goodman Cancer Institute, Metabolomics Innovation Resource, McGill University, Montreal, QC, Canada.
[7]Department of Pathology, McGill University Health Centre, Montreal, QC, Canada.
[8]Department of Pathology, IASO women's hospital, Athens, Greece.
[9]Cancer & Cell Biology Division, Translational Genomics Research Institute, Phoenix, AZ, USA
[10]Integrated Mass Spectrometry Shared Resource, City of Hope Comprehensive Cancer Center, CA, USA
[11]Translational Genomics Research Institute, Division of Integrated Cancer Genomics, Phoenix, AZ, USA
[12]Department of Pathology and Laboratory Medicine, University of North Carolina, Chapel Hill, NC, USA.
[13]Lineberger Comprehensive Cancer Center, University of North Carolina, Chapel Hill, NC, USA.
[14]Department of Medical Genetics, University of British Columbia, Vancouver, BC, Canada
[15]Department of Obstetrics and Gynaecology, University of British Columbia, Vancouver, BC, Canada
[16]Departments of Human Genetics, Medicine and Oncology McGill University, Montreal, QC, Canada.
[17]Division of Medical Genetics, Department of Specialized Medicine and Cancer Research Program, McGill University Health Centre, Montreal, QC, Canada.
[18]Division of Medical Genetics, Department of Specialized Medicine and Lady Davis Institute, Jewish General Hospital, McGill University, Montreal, QC, Canada.

* These authors contributed equally
§ These authors jointly supervised this work

Supplementary Figures 1-9

Supplementary Figure 1

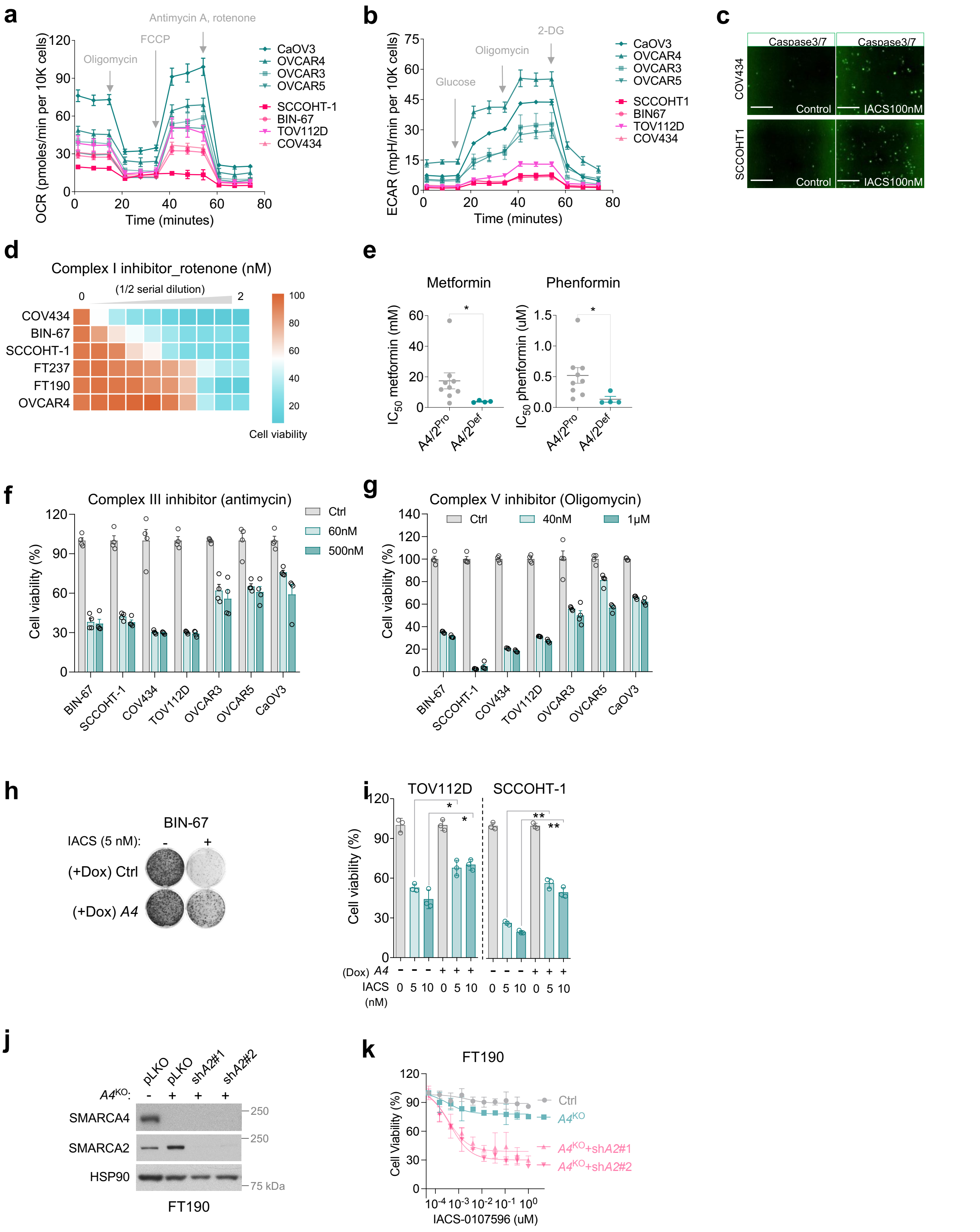

Supplementary Figure 1. *SMARCA4/2*-loss causes impaired glycolysis and increased dependence on OXPHOS. **a**, Seahorse Mito Stress Test assay measuring mitochondrial oxygen consumption rate (OCR) in a panel of ovarian cancer cell lines ($n = 5$). **b**, Seahorse Glycolysis Stress Test assay measuring extracellular acidification rate (ECAR) in a panel of ovarian cancer cell lines ($n = 6$). **c**, Representative images of SCCOHT cell lines treated with IACS-010759 (100 nM) in the presence of Caspase-3/7 Green Dye. Scale bar: 300 µm. **d**, Heatmap showing relative cell viability of indicated cell lines after being treated with different doses of Rotenone for 7 days. **e, f, g**, Responses of indicated cell lines to 3-day treatment of ETC complex inhibitors: (**e**) Metformin, Phenformin, (**f**) Antimycin and (**g**) Oligomycin. Two-tailed t-test, $n = 4$ independent experiments. p values: metformin, 0.0317; phenformin, 0.0173. **h**, Colony-formation assay in BIN-67 cells expressing doxycycline inducible *SMARCA4* upon treatment with IACS-010759 (5 nM). **i**, Cell viability of SCCOHT-1 and TOV112D cells, ± *SMARCA4* transient re-expression, treated with IACS-010759 for 3 days (two-tailed t-test, n = 3 independent experiments). p values: TOV112D, 5nM, 0.0305, 10nM, 0.01; SCCOHT-1, 5nM, 0.0021, 10nM, 0.003. **j, k** Immunoblots (**j**) and cell viability (**k**) in FT190 cells with indicated *SMARCA4/2* perturbations treated with IACS-010759 ($n = 3$ independent experiments). $^*p < 0.05$, $^{**}p < 0.01$. Error bars, mean ± SD.

Supplementary Figure 2

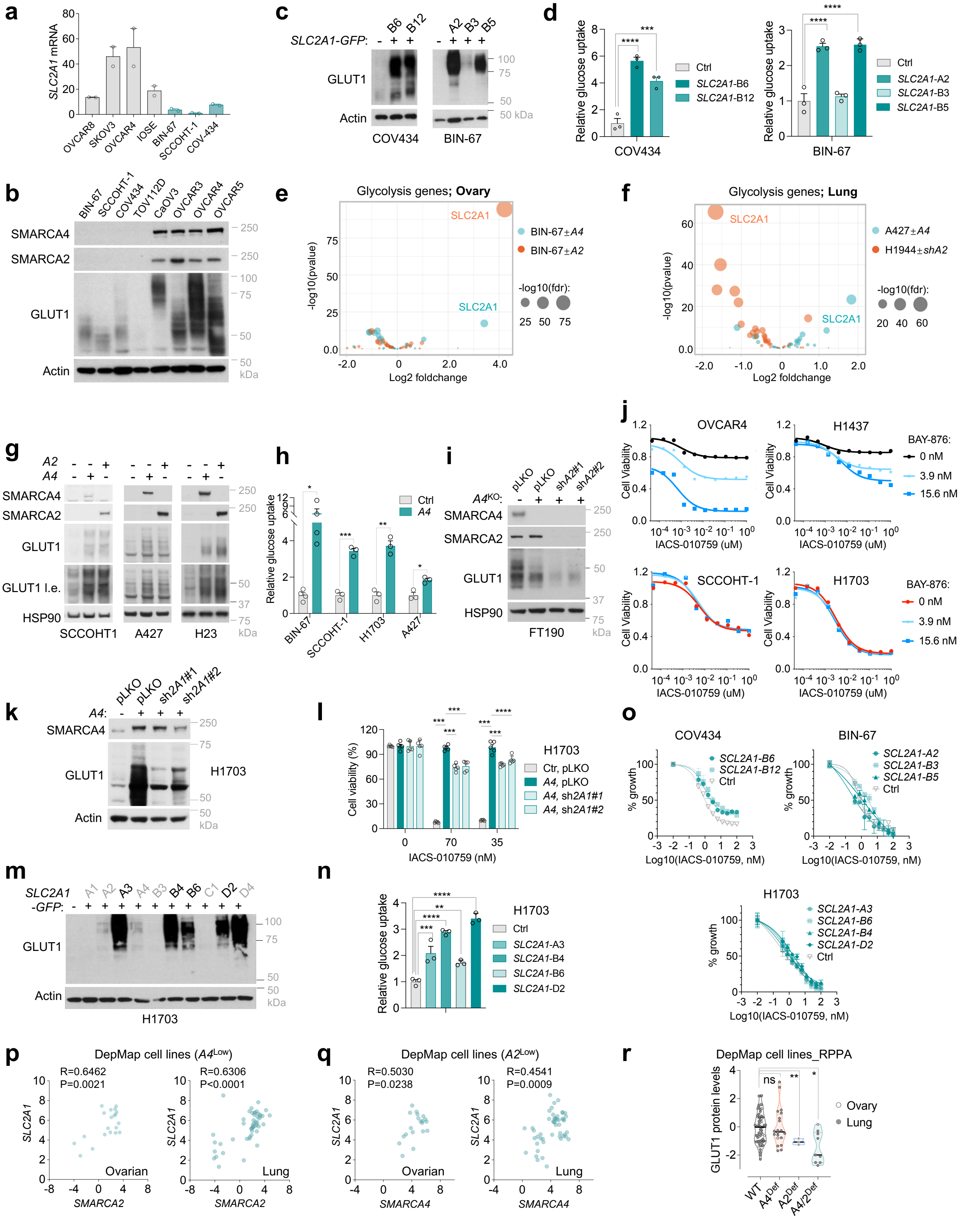

Supplementary Figure 2. *SMARCA4/2* directly regulate *SLC2A1* transcription. a, Relative *SLC2A1* mRNA expression (qRT-PCR, $n = 2$ independent experiments). **b, c**, Immunoblots of parental ovarian cancer cells (**b**) and COV-434 and BIN-67 cells ± *SCL2A1-GFP* overexpression (**c**). **d**, Glucose-uptake capacity in cells described in (**c**), $n = 3$ independent experiments. p values: COV434, B6, < 0.0001, B12, 0.0006; BIN-67, < 0.0001. **e, f**, Volcano plot of differentially expressed glycolysis genes in ovarian (**e**) and lung (**f**) cancer cell lines ± *SMARCA4/2* (A4, A2) restoration or *SMARCA2* knockdown (sh*A2*). Wald test (DEseq2). **g,** Immunoblots of cell lines ± *SMARCA4/2* restoration. **h**, Glucose-uptake capacity in cell lines ± *SMARCA4* restoration (BIN-67, $n = 4$; others, $n = 3$ independent experiments). p values: BIN-67, 0.0458; SCCOHT-1, 0.0003; H1703, 0.0019; A427, 0.0132. **i,** Immunoblots of FT190 cells ± *SMARCA4/2* perturbations. **j,** Cell viability with the treatment of IACS-010759 ± BAY-876. **k, l**, Immunoblots (**k**) and cell viability (**l**) of H1703 expressing doxycycline-inducible *SMARCA4* (*A4*) ± pLKO control or shRNAs targeting *SLC2A1* (sh*2A1*), $n = 5$ independent experiments. **m**, **n**, Immunoblots (**m**) and glucose-uptake capacity (**n**) of H1703 cells ± ectopic *SCL2A1-GFP* expression, $n = 3$ independent experiments. p values: A3, 0.0008; B4, < 0.0001; B6, 0.013; D2, < 0.0001. **o**, Growth responses of BIN-67 and H1703 cells ± *SCL2A1-GFP* overexpression, $n = 4$ independent experiments. **p, q**, Correlation of *SLC2A1* and *SMARCA2* mRNA in ovarian (n = 20, p = 0.0021) and lung (n = 50, p<0.0001) cancer cell lines expressed low *SMARCA4* (**p**); correlation of *SLC2A1* and *SMARCA4* mRNA in ovarian (n = 20, p = 0.0238) and lung (n = 50, p = 0.0009) cancer cell lines expressed low *SMARCA2* (**q**). Expression data were obtained from Cancer Cell Line Encyclopedia. $A4^{Low}/A2^{Low}$, bottom quartile. R: Pearson correlation, two-tailed. **r**, Violin plot showing GLUT1 protein levels in ovarian and lung cancer cell lines using proteomics data from DepMap. p value: $A4/2^{def}$, 0.0031; $A2^{def}$, 0.029. Each dot denotes a cell line. **d, l**, **n**, **r**, One-way ANOVA corrected for multiple comparisons. **h**, two-tailed t-test. *$p < 0.05$, **$p < 0.01$, ***$p < 0.001$, ****$p < 0.0001$. ns, not significant. Error bars, mean ± SD.

Supplementary Figure 3

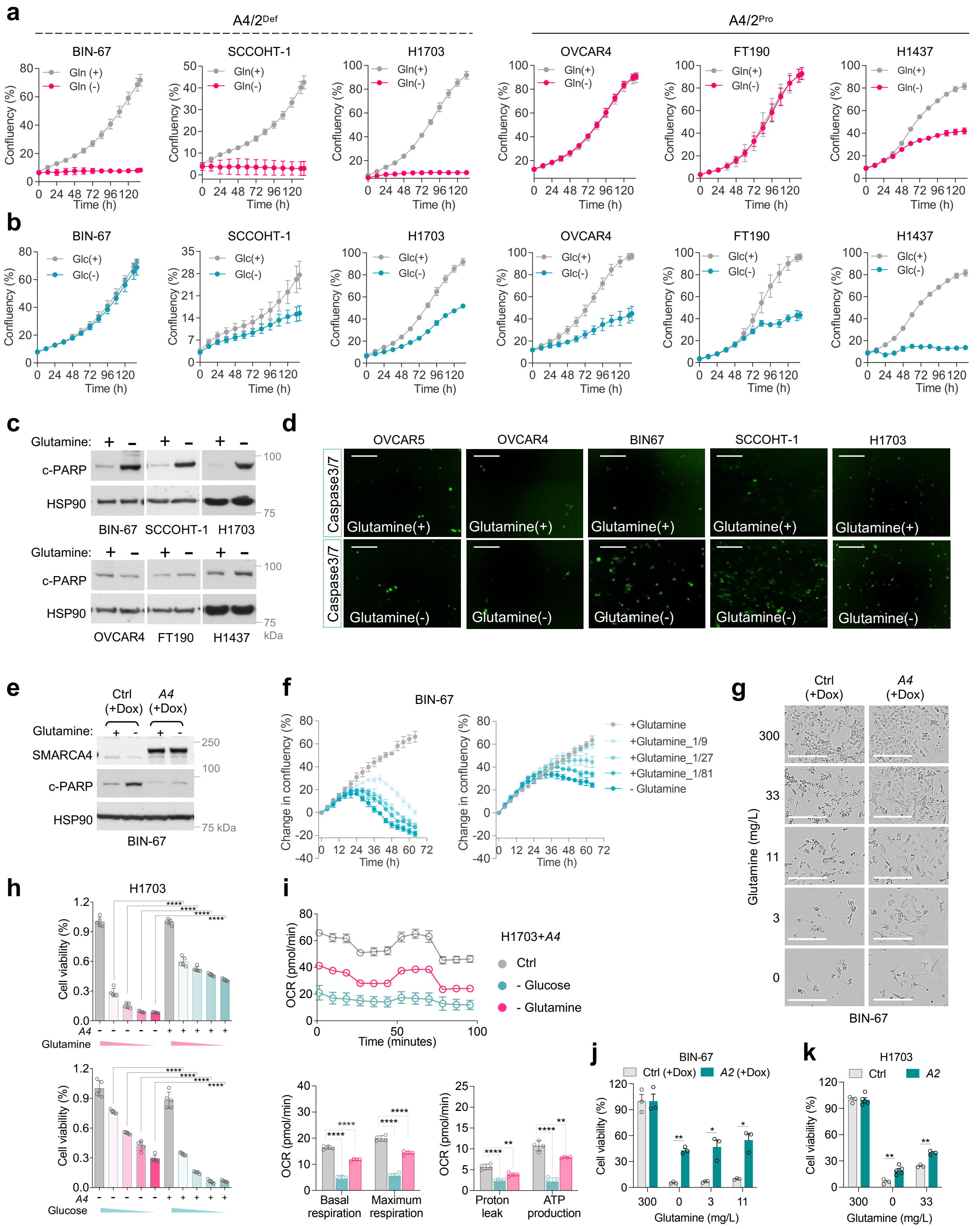

Supplementary Figure 3. ***SMARCA4/2*****-deficient cancer cells rely on glutamine instead of glucose.** **a, b**, Growth curves measured by Incucyte live cell imaging analyses of *A4/2*^{Def} and *A4/2*^{Pro} cell lines cultured ± glutamine (**a**) and glucose (**b**) for indicated times ($n = 4$ independent experiments). **c**, Immunoblots analysis of indicated cell lines cultured ± r 3 days. **d**, Representative image of indicated cell lines cultured with or without glutamine for 3 days in the presence of Caspase-3/7 Green Dye. scale bar: 300 μm. **e**, Immunoblots analysis of indicated cell lines cultured with or without glutamine for 3 days in BIN-67 cells expressing doxycycline (dox)-inducible SMARCA4. **f**, **g**, Growth curves (**f**) and representative phase-contrast images (**g**) of BIN-67 cells expressing dox inducible *SMARCA4* cultured with different concentration of glutamine ($n = 4$ independent experiments). scale bar: 150 μm. **h**, Cell viability of H1703 cells with *SMARCA4* restoration cultured with different concentration of glutamine (top; 300, 33, 11, 3.3, 0 mg/L) or glucose (bottom; 2000, 220, 74, 25, 0 mg/L) for 4 days. Two-tailed t-test, $n = 5$ independent experiments. p value < 0.0001. **i**, Seahorse mito stress test assay measuring mitochondrial OCR in H1703 cells with *SMARCA4* restoration in the absence of glucose or glutamine (Top). Basal respiration, maximum respiratory capacity, proton leak and ATP production were computed in H1703 cells with *SMARCA4* restoration cultured with or without glucose or glutamine (Bottom). Ctrl $n = 4$, -Glucose, $n = 5$, -Glutamine, $n = 4$ independent experiments. p values: -Glutamine, proton, 0.0014, ATP, 0.002; others < 0.0001). **j, k**, Cell viabilities of BIN-67 cells -/+ doxycycline-inducible SMARCA2 re-expression (**j**) or H1703 cells with *SMARCA2* restoration (**k**), cultured with different concentration of glutamine. BIN-67, $n = 3$; H170+A2, 300mg/L, $n = 5$, 0mg/L, $n = 5$; others: $n = 3$. p values: BIN-67, 0mg/L, 0.002, 3mg/L, 0.0361, 11mg/L, 0.0247; H1703, 0mg/L, 0.0029, 33mg/L, 0.0011. $n = 3$ independent experiments. **h**, **i**, One-way ANOVA corrected for multiple comparisons. **j**, **k**, Two-tailed t-test. *$p < 0.05$, **$p < 0.01$, ***$p < 0.001$, ****$p < 0.0001$. Error bars, mean ± SD.

Supplementary Figure 4

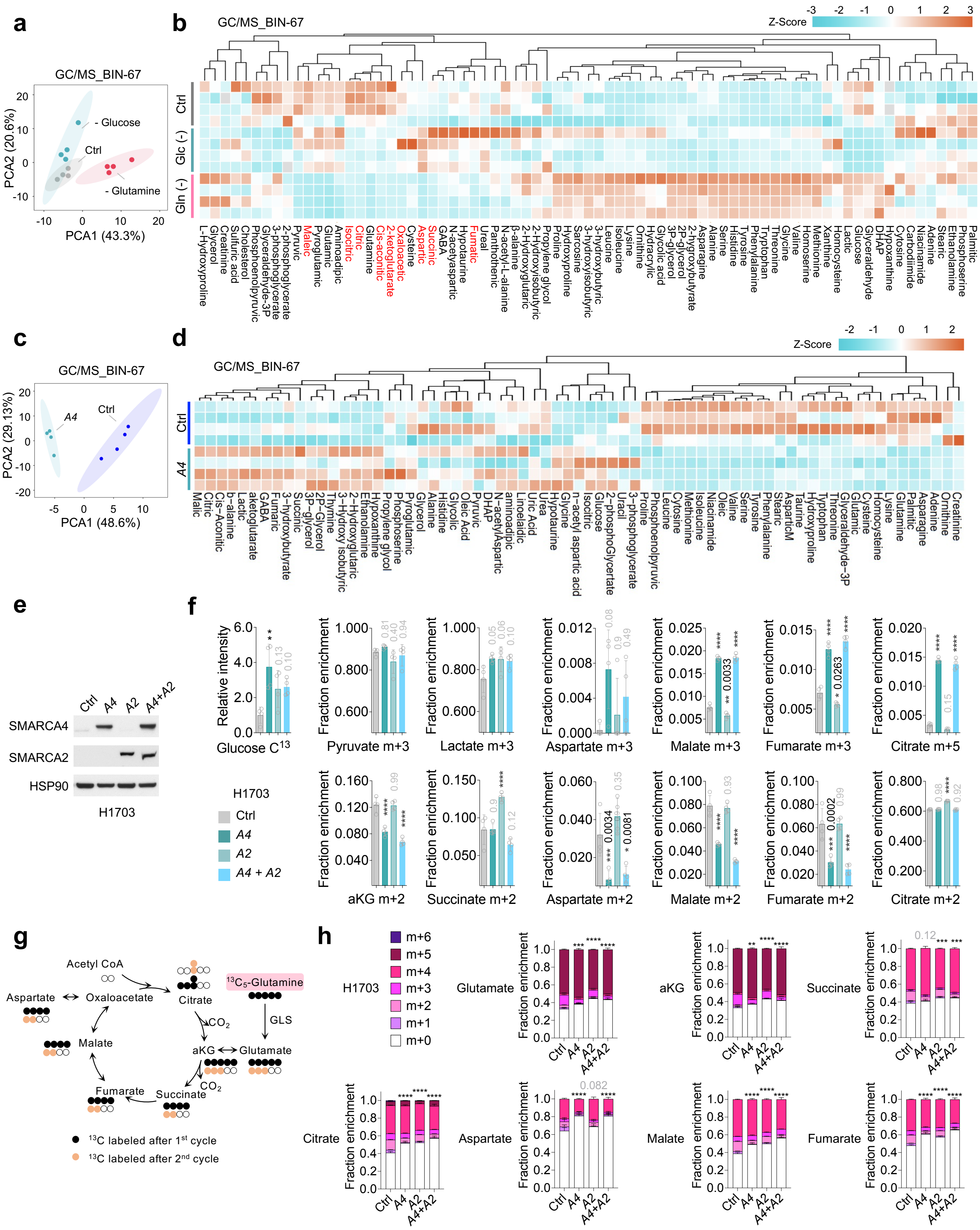

Supplementary Figure 4. *SMARCA4* restoration drives the metabolic shift using glucose instead of glutamine in *SMARCA4/2*-deficient cancer cells. **a**, PCA analysis of metabolite profiles of BIN-67 cells cultured with or without glucose or glutamine for 24 hours. **b**, Heatmap showing the abundance of different metabolites in BIN-67 cells cultured with or without glucose or glutamine for 24 hours. Z-score was used to compare the levels of each metabolite between different groups, which describes value of each group above or below the mean value. TCA cycle intermediates are highlighted in red. **c**, PCA analysis of metabolite profiles of BIN-67 cells ± *SMARCA4* restoration for 24 hours. **d**, Heatmap showing the abundance of different metabolites in BIN-67 cells ± *SMARCA4* restoration. **e**, Immunoblots of H1703 cells with or without SMARCA4 (A4) or/and SMARCA2 (A2) restoration. **f**, Relative total labelled $^{13}C_6$-glucose and fractional isotopic incorporation of $^{13}C_6$-glucose in H1703 cells ± *SMARCA4* or/and SMARCA2 restoration (into indicated intermediates measured by GC/MS. Cells were cultured in $^{13}C_6$-glucose containing medium for 30 mins before harvesting for GC/MS ($n = 4$ independent experiments). **g**, Diagram of stable isotope tracer analysis using uniformly labelled $^{13}C_5$-glutamine. Solid circle, ^{13}C; open circle, ^{12}C. **h**, Fractional isotopic incorporation of $^{13}C_5$-glutamine into TCA cycle intermediates were measured by GC/MS in H1703 cells ± *SMARCA4/2* restoration, cultured in $^{13}C_5$-glutamine containing medium for 1-hour ($n = 4$ independent experiments). p values: glutamine, A4, 0.0003; a-KG, A4, 0.0027; succinate, A4, 0.1209, A2, 0.0004, A4+A2, 0.0004; aspartate, A2, 0.082; others < 0.0001. m, number of labelled carbons. **f**, **h**, One-way ANOVA corrected for multiple comparisons. *$p < 0.05$, **$p < 0.01$, ***$p < 0.001$, ****$p < 0.0001$. Error bars, mean ± SD.

Supplementary Figure 5

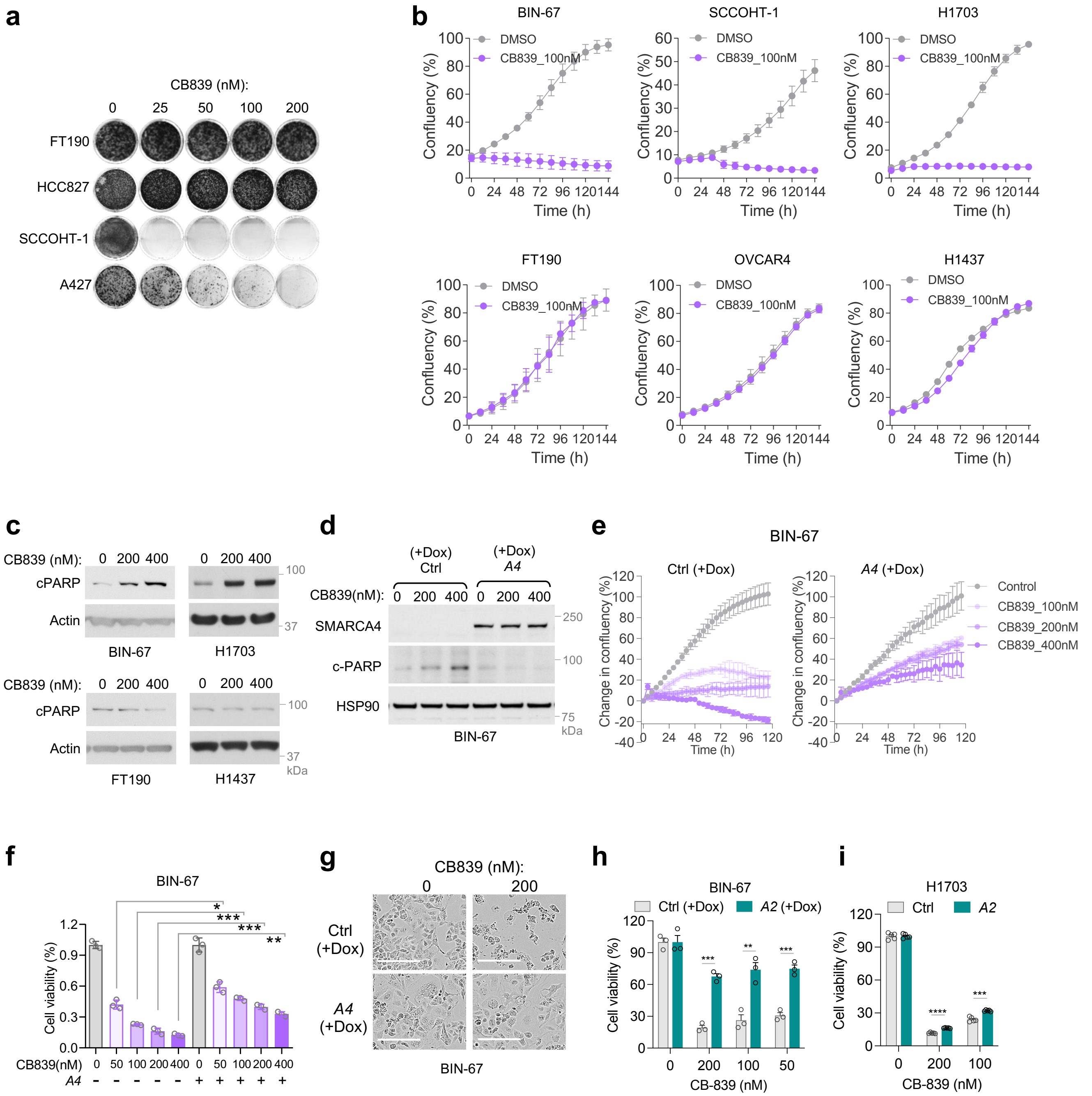

Supplementary Figure 5. ***SMARCA4/2*** **restoration rescues glutamine dependency in** ***SMARCA4/2*****-deficient cancer cells.**

a, Colony-formation assay of the indicated cell lines cultured in the presence of different concentrations of CB839 for 10-14 days. **b**, Growth curves measured by Incucyte live cell analyses of indicated cell lines cultured with or without CB839 (100nM) for indicated time, $n = 3$ independent experiments. **c**, Immunoblots of indicated cancer cell lines cultured with different concentrations of CB839 for 3 days. **d**-**g**, Immunoblots (**d**), growth curves (**e**), cell viability (**f**) and representative phase-contrast images (**g**) of BIN-67 cells expressing doxycycline (dox)-inducible SMARCA4 (A4) cultured with different doses of CB839. p values: 50nM, 0.013; 100nM, 0.0004; 200nM, 0.0005; 400nM, 0.0012. scale bar: 300 µm. $n = 3$ independent experiments. **h**, Cell viabilities of BIN-67 cells, -/+ doxycycline-inducible SMARCA2 (A2) re-expression, cultured with different concentration of CB839. p values: 200nM, 0.0002; 100nM, 0.0059; 50nM, 0.0008. $n = 3$ independent experiments. **i,** Cell viability of H1703 cells with *SMARCA2* restoration cultured with different concentration of CB839. p values: 200nM, < 0.0001; 100nM, 0.0006. $n = 5$ independent experiments**.** **f**, **h**, **i**, Two-tailed t-test. $*p < 0.05$, $**p < 0.01$, $***p < 0.001$, $****p < 0.0001$. Error bars, mean ± SD. A4, SMARCA4; A2, SMARCA2; Dox, doxycycline.

Supplementary Figure 6

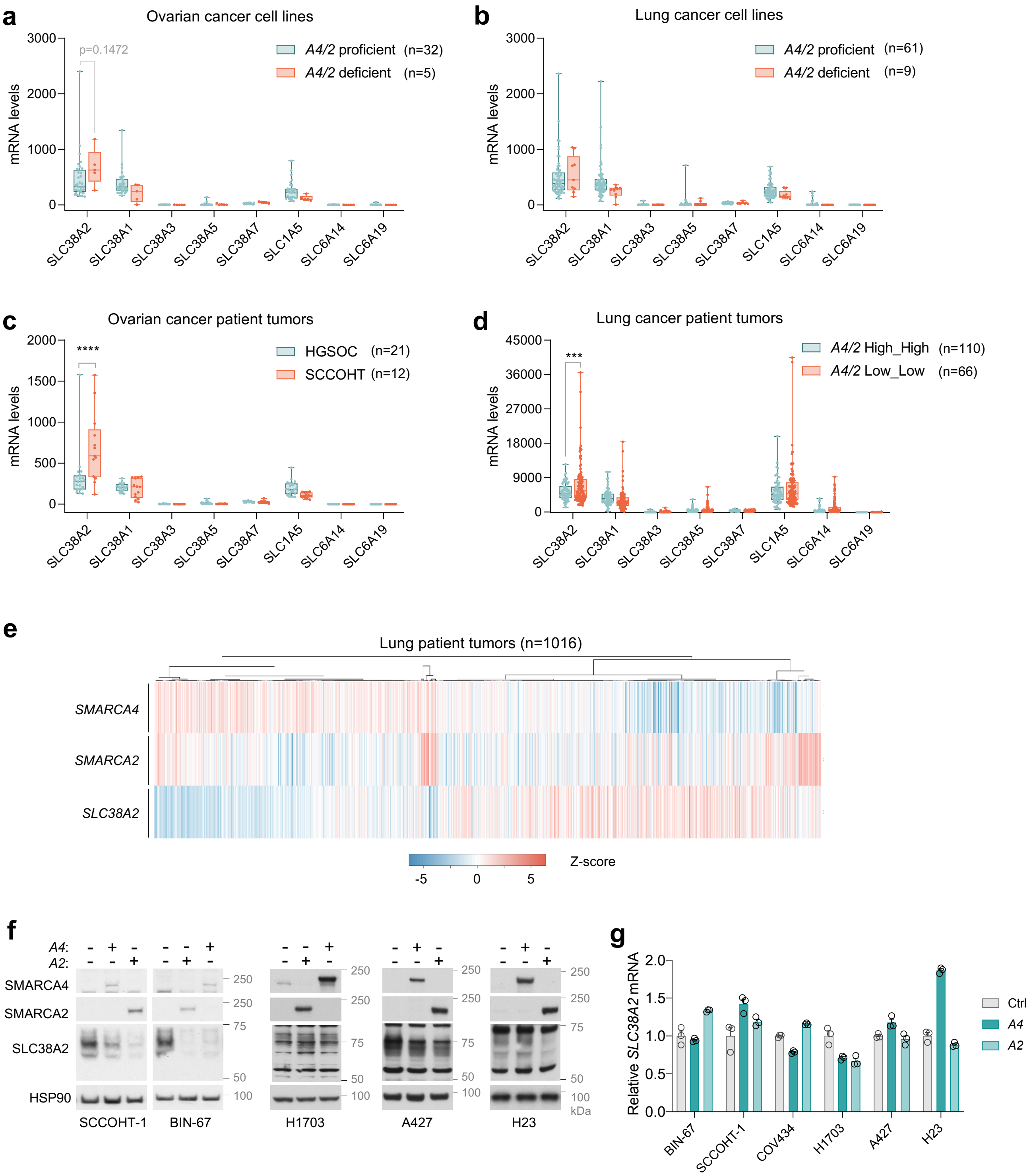

Supplementary Figure 6. *SMARCA4/2*-loss leads to elevated *SLC38A2* expression. a, Box plot showing mRNA levels of indicated glutamine transporters in *SMARCA4/2*-proficient (n = 32) and -deficient (n = 5) ovarian cancer cell lines. **b**, Box plot showing indicated glutamine transporter mRNA levels in *SMARCA4/2*-proficient (n = 61) and deficient (n = 9) lung cancer cell lines. **c**, Box plot showing mRNA levels of indicated glutamine transporters in HGSOC (n = 21) and SCCOHT (n = 12) patient tumors, p value < 0.0001. **d**, Box plot showing mRNA levels of indicated glutamine transporters in *SMARCA4/2*-high (n = 110) and *SMARCA4/2*-low (n = 66) lung cancer samples. p value = 0.0002. **e**, Heatmap showing *SMARCA4*, *SMARCA2* and *SLC38A2* mRNA levels in lung cancer patient tumor samples (n = 1016). The patient samples were arranged by supervised complete-linkage hierarchical clustering using the Pearson distance as the distance measure. **f**, **g**, Protein and mRNA levels of in indicated cell lines ± *SMARCA4/2* restoration measured by immunoblotting (**f**) and RT-qPCR (**g**). $n = 3$ independent experiments. (**a-d**) Two-way ANOVA analysis followed by Sidak's multiple comparisons test. In each box plot, the upper box border represents the 75th quartile, the lower box border represents the 25th quartile, with the center line depicting the median and whiskers representing the range. ***p < 0.001, ****p < 0.0001. A4, SMARCA4; A2, SMARCA2.

Supplementary Figure 7

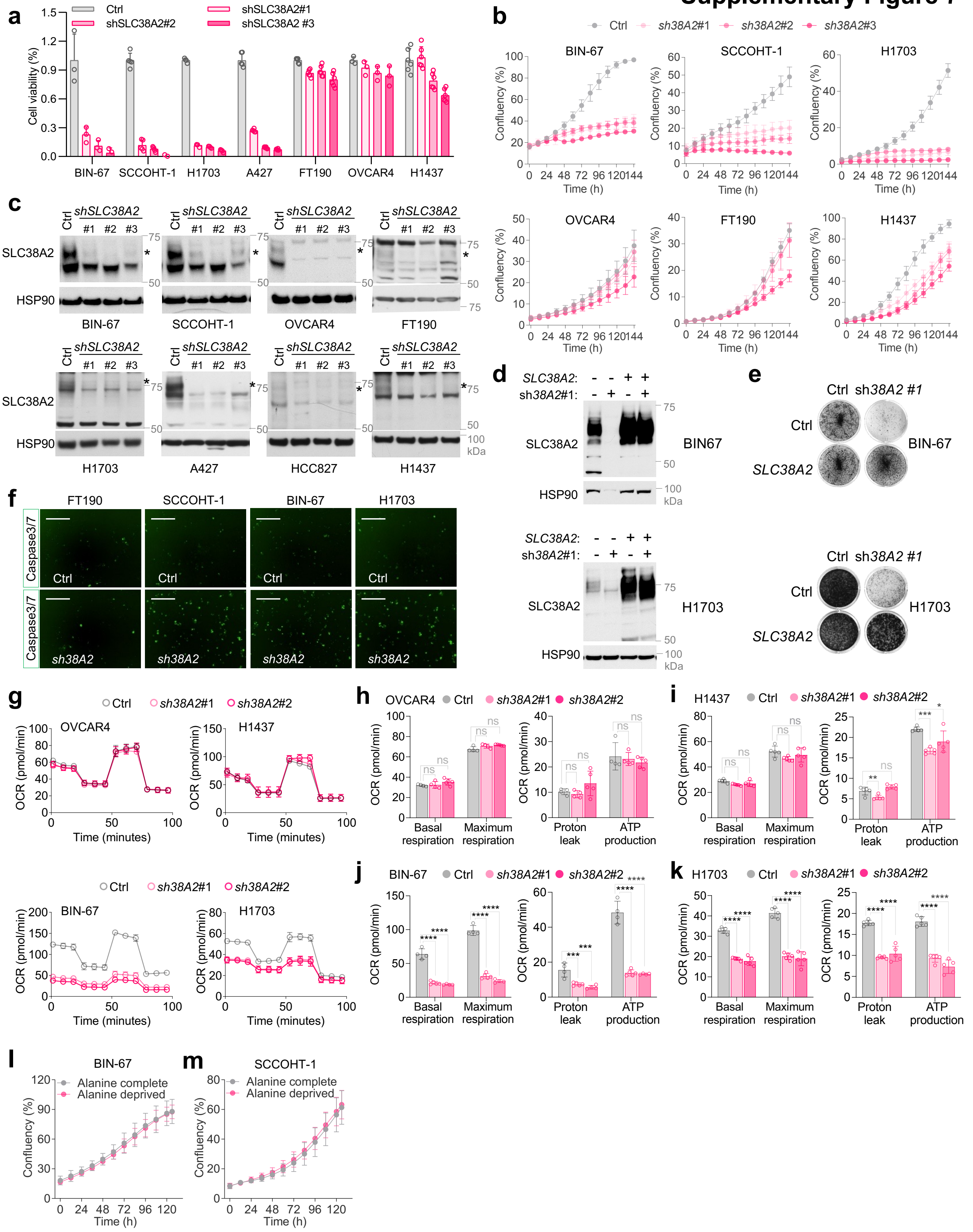

Supplementary Figure 7. ***SMARCA4/2*-deficient cancer cells rely on elevated glutamine transporter *SLC38A2*.** **a**, **b**, Cell viability (**a**) and growth curves (**b**) of indicated cell lines expressing pLKO control or shRNAs targeting *SLC38A2* after culturing for 7 days (**a**) and indicated times (**b**). BIN-67, $n = 3$; SCCOHT-1, $n = 5$; H1703, $n = 4$; A427, $n = 6$; FT190, $n = 6$; OVCAR4, $n = 3$; H1437, $n = 6$ independent experiments. **c**, Immunoblots of indicated cell lines expressing pLKO control or shRNAs targeting *SLC38A2* (sh*38A2*). Asterisks indicate the dominant SCL38A2 bands in each cell line. **d**, **e**, Immunoblots (**d**) and colony formation assays (**e**) of BIN-67 and H1703 cells expressing pLKO control or shRNA#1 targeting the 3'UTR of *SLC38A2*, ± restoration of the *SCL38A2* cDNA lacking the 3'UTR. **f**, Representative images of indicated cells lines expressing pLKO control or shRNAs targeting *SLC38A2* cultured in medium containing Caspase-3/7 Green Dye. scale bar: 300 µm. **g**, Seahorse Mito Stress Test assay measuring mitochondrial OCR in indicated cell lines expressing pLKO control or shRNAs targeting *SLC38A2*. **h-k**, Effect of *SLC38A2* knockdown on mitochondrial respiration. Basal respiration, maximum respiratory capacity (maximum respiration), proton leak and ATP production were computed from OCR measurements (**g**) in OVCAR4 (**h**), H1437 (**i**), BIN-67 (**j**) and H1703 (**k**) cells expressing pLKO control or shRNAs targeting *SLC38A2.* OVCAR4, Ctrl, $n = 4$, sh#1, $n = 4$, sh#2, $n = 5$; H1437, $n = 5$; BIN-67, Ctrl, $n = 4$, sh#1, $n = 5$, sh#2, $n = 4$; H1703, $n = 5$ independent experiments. One-way ANOVA corrected for multiple comparisons. p values: H1437, proton sh*38A2*#1, 0.0083, ATP sh*38A2*#1, 0.0004, ATP sh*38A2*#2, 0.0202; BIN-6, proton sh*38A2*#1, 0.0008, proton sh*38A2*#2, 0.0003, others < 0.0001; H1703, all < 0.0001. **l**, **m**, Growth curves measured by Incucyte live cell imaging analyses in BIN-67 (**l**) and SCCOHT-1 (**m**) cells cultured in RMPI containing dialyzed FBS ± alanine (13.4 mg/L) for indicated times ($n = 2$ independent experiments). $^{**}p < 0.01$, $^{***}p < 0.001$, $^{****}p < 0.0001$. Error bars, mean ± SD.

Supplementary Figure 8

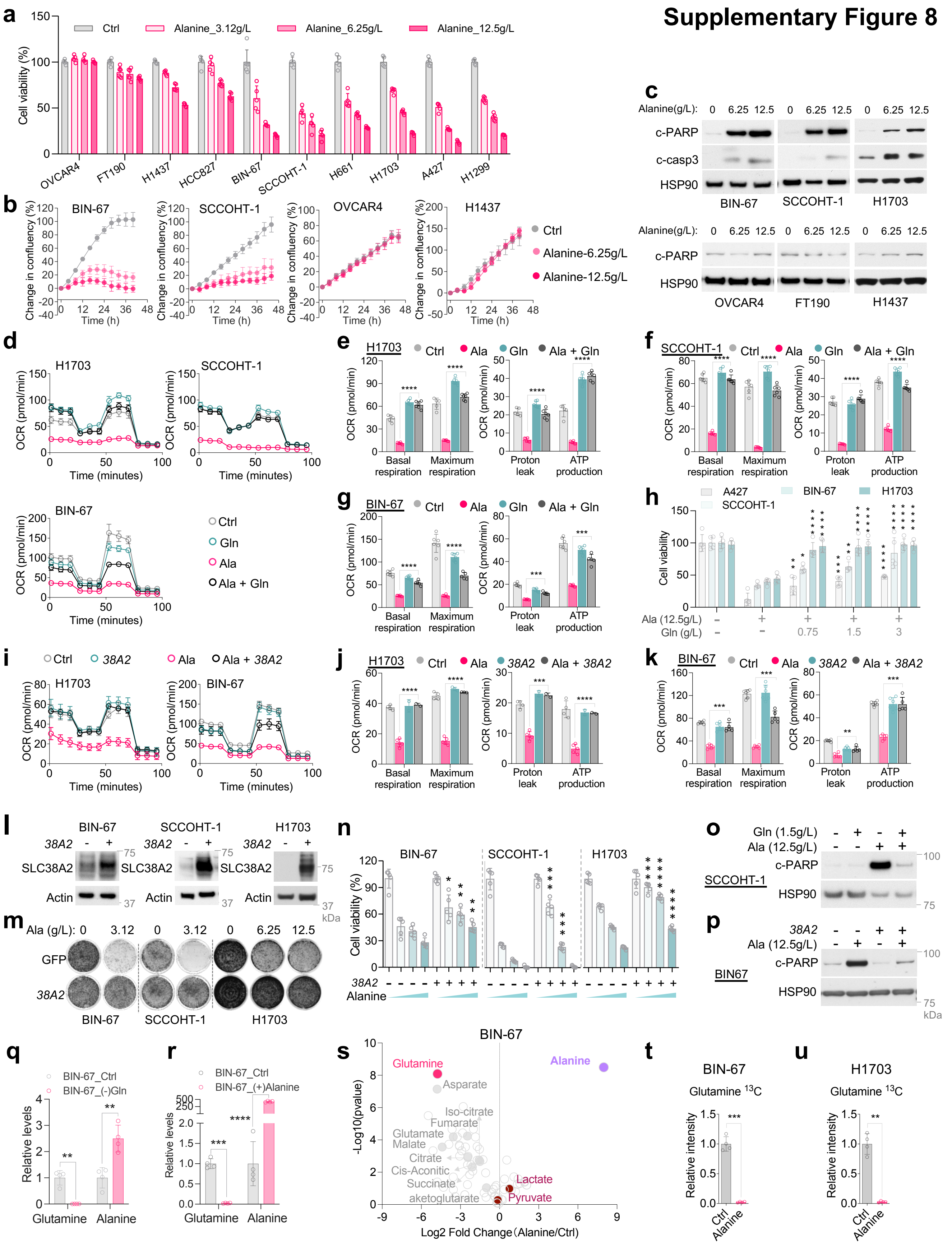

Supplementary Figure 8. Alanine supplementation suppresses *SMARCA4/2*-deficient cancer cells through competing with glutamine for SLC38A2. **a**, **b**, **c**, Cell viability (**a,** 7 days), growth curves (**b**) (FT190: $n = 6$; other cell lines: $n = 5$ independent experiments) and immunoblots (**c**, 3 days) of cell lines cultured with alanine supplementation. **d**, Seahorse assay measuring mitochondrial OCR in cell lines cultured ± alanine (Ala) BIN-67 and SCCOHT-1, 6.25 g/L; H1703, 12.5 g/L) or glutamine (Gln) (1.5 g/L). **e-g**, Basal respiration, maximum respiratory, proton leak and ATP production were computed from OCR data (**d**) for H1703 (**e**), SCCOHT-1 (**f**) and BIN-67 (**g**). H1703, Ctrl, $n = 5$, Ala, $n = 6$, Gln, $n = 6$, Ala + Gln, $n = 6$; SCCOHT-1, Ctrl, $n = 6$, Ala, $n = 5$, Gln, $n = 6$, Ala + Gln, $n = 6$; H1437, $n = 5$; BIN-67, Ctrl, $n = 6$, Ala, $n = 6$, Gln, $n = 6$, Ala + Gln, $n = 5$ independent experiments. p values: H1703, SCCOHT-1, all < 0.0001; BIN-67, proton, 0.0004, ATP, 0.0002; others < 0.0001. **h**, Cell viability of cell lines cultured ± alanine or glutamine for 5 days ($n = 5$ independent experiments). p values: A427, Gln 0.75, 0.0051, Gln 1.5, 0.0004, Gln-3, < 0.0001; SCCOHT-1, Gln 0.75, 0.0178, Gln 1.5, 0.0068, Gln-3, < 0.0001; BIN-67, all < 0.0001; H1703, all < 0.0001. **i,** Seahorse assay measuring mitochondrial OCR in cell lines ± *SLC38A2* (*38A2*) overexpression cultured ± alanine (BIN-67, 6.25 g/L; H1703, 12.5 g/L). **j, k**, Basal respiration, maximum respiratory, proton leak and ATP production were computed from OCR data (**i**) for H1703 (**j**) and BIN-67 (**k**) cells ± *SLC38A2* overexpression cultured ± alanine H1703, Ctrl, $n = 4$, Ala, $n = 5$, 38A2, $n = 3$, Ala + 38A2, $n = 2$; BIN-67, Ctrl, $n = 6$, Ala, $n = 6$, 38A2, $n = 6$, Ala + 38A2, $n = 5$ independent experiments. p values: H1703, proton, 0.0002, others, < 0.0001; BIN-67, basal, 0.0003, maximum, 0.0002, proton, 0.0032, ATP, 0.0002. **l**, Immunoblots. **m**, Colony-formation assay of cell lines ± *SLC38A2* overexpression treated with alanine for 10–15 days. **n**, Cell viability of indicated cell lines ± *SLC38A2* overexpression treated with alanine (3.125 g/L, 6.25 g/L and 12.5 g/L) for 5 days ($n = 5$). p values, from left to right: BIN-67, 0.0319, 0.0033, 0.0011; SCCOHT-1, 0.0002, 0.0002; H1703 0.0002, < 0.0001, < 0.0001. **o, p**, Immunoblots of SCCOHT-1 cells cultured ± glutamine or alanine (**o**) and in BIN-67 cells ± *SLC38A2* overexpression cultured with alanine (**p**) for 3 days. **q**, Relative abundance of glutamine and alanine measured by GC/MS in BIN-67 cells after culturing without glutamine for 24 hours ($n = 4$). p values: left, 0.0055; right, 0.0041. **r**, Relative abundance of glutamine and alanine measured by GC/MS in BIN-67 cells cultured with alanine (6.25 g/L) for 14 hours ($n = 4$). p values: left, 0.0005; right, < 0.0001. **s**, Differential abundance of metabolites measured by GC/MS in BIN-67 cultured with alanine (6.25 g/L) for 14 hours. Two-tailed t-test. **t, u** Relative total labelled $^{13}C_5$-glutamine in BIN-67 (**t**) and H1703 (**u**) cells after incubating with $^{13}C_5$-glutamine containing medium for 1 hour. Cells were pretreated with alanine (6.25 g/L) for 14 hours ($n = 4$). p values BIN-67, 0.0005; H1703, 0.0015). (**e-g, j, k, n, q, r, t, u**) Two-tailed t-test. **h**, One-way ANOVA corrected for multiple comparisons. $^{**}p < 0.01$, $^{***}p < 0.001$, $^{****}p < 0.0001$. Error bars, mean ± SD.

Supplementary Figure 9

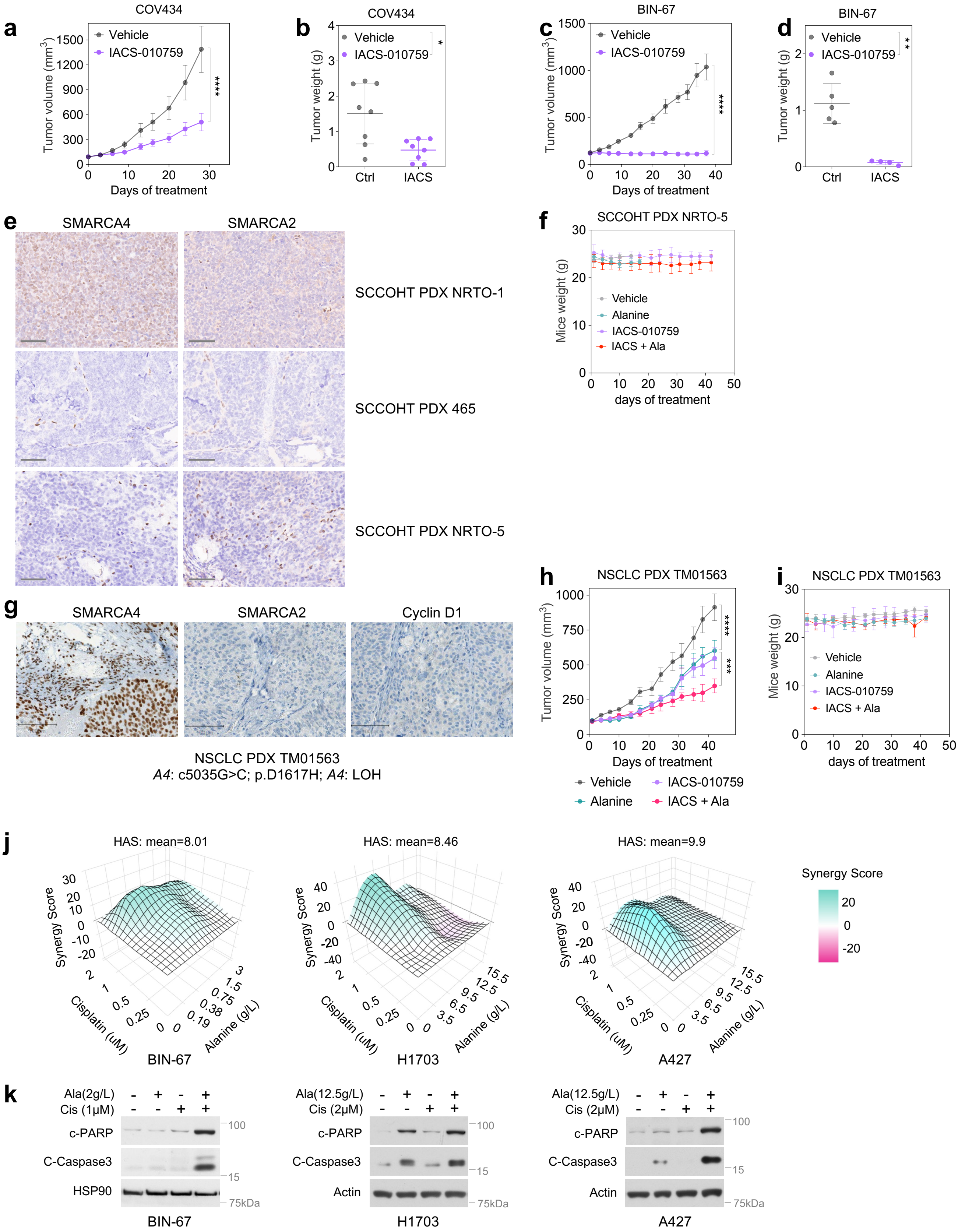

Supplementary Figure 9. Inhibitors targeting OXPHOS or glutaminase, and alanine supplementation-based treatments suppress the growth of *SMARCA4/2*-deficient tumors. **a**, **b** Tumor volumes (**a**) and final tumor weights (**b**) of mice injected with COV434 cells after treating with vehicle or IACS-010759 (7.5 mg/kg, n = 8) for indicated times. *p* values: **a**, < 0.0001; **b**, 0.0112. **c**, **d** Tumor volumes (**c**) and final tumor weights (**d**) of mice injected with BIN-67 cells after treating with vehicle (n = 5) or IACS-010759 (7.5 mg/kg, n = 4) for indicated times. *p* values: **a**, < 0.0001, **b**, 0.0026. **e-i**, Treatment validation using patient derived xenograft (PDX) tumors. **e**, **g**, Representative images of immunohistochemistry (IHC) analysis were performed on formalin-fixed paraffin embedded SCCOHT (**e**) and NSCLC (**g**) PDX tumors with indicated protein markers. All SCCOHT PDX models were generated from clinically confirmed SCCOHT patient tumors The NSCLC PDX obtained from The Jackson Laboratory (TM01563) harbored a missense mutation (D1617H) and LOH (CNV, 0.38) in *SMARCA4* gene. Loss of cyclin D1 expression, a direct target of SMARCA4/2 (PMID: 30718512), was used to verify that the retained SMARCA4 was loss of function. Scale bar: 60 μm. **f**, **i**, Weight of mice bearing tumors of SCCOHT PDX NRTO-5 (**f**) and NSCLC PDX TM01563 (**i**) treated with vehicle, IACS-010759 (IACS, 7.5 mg/kg), alanine (ala, 4 g/kg) or their combination (IACS + Ala). **h**, Tumor volumes of mice bearing NSCLC PDX tumors treated with vehicle, IACS-010759 (7.5 mg/kg), alanine (4 g/kg) or their combination (n = 4 for each arm). *p* values: alanine vs IACS + Ala, 0.0007; vehicle vs IACS + Ala, < 0.0001. **j**, Synergy score plots of alanine plus cisplatin combination in indicate cell lines calculated by SynergyFinder. **k**, Immunoblotting analysis of cell apoptosis in indicated cell lines treated with or without alanine or cisplatin. (**a, c, h**) Two-way ANOVA. (**b, d**) Two-tailed t-test. *$p < 0.05$, **$p < 0.01$, ****$p < 0.0001$. Error bars, mean ± SEM.
