# Supplementary material for: Alanine supplementation exploits glutamine dependency induced by SMARCA4/2-loss
Source: Nat Commun. 2023 May 20;14:2894. doi: 10.1038/s41467-023-38594-3 (PMC10199906; doi:10.1038/s41467-023-38594-3)
Supplement: Supplementary file 2 — Reporting Summary [file 41467_2023_38594_MOESM2_ESM.pdf]

nature portfolio

Corresponding author(s): Yemin Wang and Sidong Huang

Last updated by author(s): Apr 26, 2023

Reporting Summary

Nature Portfolio wishes to improve the reproducibility of the work that we publish. This form provides structure for consistency and transparency in reporting. For further information on Nature Portfolio policies, see our Editorial Policies and the Editorial Policy Checklist.

Statistics

For all statistical analyses, confirm that the following items are present in the figure legend, table legend, main text, or Methods section.

n/a	Confirmed	
☐	☒	The exact sample size (n) for each experimental group/condition, given as a discrete number and unit of measurement
☐	☒	A statement on whether measurements were taken from distinct samples or whether the same sample was measured repeatedly
☐	☒	The statistical test(s) used AND whether they are one- or two-sided<br>*Only common tests should be described solely by name; describe more complex techniques in the Methods section.*
☒	☐	A description of all covariates tested
☐	☒	A description of any assumptions or corrections, such as tests of normality and adjustment for multiple comparisons
☐	☒	A full description of the statistical parameters including central tendency (e.g. means) or other basic estimates (e.g. regression coefficient) AND variation (e.g. standard deviation) or associated estimates of uncertainty (e.g. confidence intervals)
☐	☒	For null hypothesis testing, the test statistic (e.g. F, t, r) with confidence intervals, effect sizes, degrees of freedom and P value noted<br>*Give P values as exact values whenever suitable.*
☒	☐	For Bayesian analysis, information on the choice of priors and Markov chain Monte Carlo settings
☒	☐	For hierarchical and complex designs, identification of the appropriate level for tests and full reporting of outcomes
☐	☒	Estimates of effect sizes (e.g. Cohen's d, Pearson's r), indicating how they were calculated

Our web collection on statistics for biologists contains articles on many of the points above.

Software and code

Policy information about availability of computer code

Data collection	1. Caspase3/7 and phase contrast images and cell growth curve: IncuCyte® S3 Software (v2016B)<br>2. Western blots and colony formation images: Epson Perfection V700 Photo (Epson)<br>3. RT-qPCR: QuantStudio5 (ThermoFisher)<br>4. IHC images: Aperio Scanscope XT (Leica Biosystems), Lumenera INFINITY X CMOS Camera (Teledyne Lumenera)<br>5. Mitochondrial respiration and glycolysis measurements: XFe96 or XFe24 Analyzer (Seahorse Bioscience)<br>6. Metabolite profiling and isotope tracing: GC/MS instrument (5975C, Agilent)<br>7. Glucose uptake: InfiniteM200Pro (Tecan)<br>7. Cell viability Assay: InfiniteM200Pro (Tecan)<br>8. Heatmap: pheatmap package in R (4.1.1)
Data analysis	1. Mitochondrial respiration and glycolysis analysis: Wave 2.6 software (Agilent, V2.4.3.3.3)<br>2. Metabolite profiling and isotope tracing: Mass Hunter Quant software (Agilent, V10.2.733.8)<br>3. Statistics and graphing: GraphPad Prism 8 (V8.4.0)<br>4. Transcriptome analysis: STAR (2.6.1c), Homer (v4.1.1), Samtools (v1.3.1), DESeq2 (1.19.38), R (4.1.1), RStudio (1.4.1717)<br>5. Caspase3/7 images: IncuCyte® S3 Software (v2016B)<br>6. IHC image analysis: Aperio ImageScope (12.3.3)

For manuscripts utilizing custom algorithms or software that are central to the research but not yet described in published literature, software must be made available to editors and reviewers. We strongly encourage code deposition in a community repository (e.g. GitHub). See the Nature Portfolio guidelines for submitting code & software for further information.

Data

Policy information about availability of data

All manuscripts must include a data availability statement. This statement should provide the following information, where applicable:

- Accession codes, unique identifiers, or web links for publicly available datasets
- A description of any restrictions on data availability
- For clinical datasets or third party data, please ensure that the statement adheres to our policy

Protein and mRNA data of different cancer cell lines are obtained from the DepMap (https://depmap.org/portal/). The mRNA expression data of indicated genes in ovarian cancer patients was downloaded from UCSC Xena (https://xenabrowser.net/datapages/). The mRNA expression data of indicated genes in lung cancer patients were obtained from cBioportal (https://www.cbioportal.org/). RNA-seq data of 13 SCCOHT patient tumors were obtained from previous studies (PMID: 26343384 and PMID: 34518526). Source data for RNA-seq, ChIP-seq and ATAC-seq can be found using the accession number GSE151026 [https://www.ncbi.nlm.nih.gov/geo/query/acc.cgi?acc=GSE151026], GSE117735 [https://www.ncbi.nlm.nih.gov/geo/query/acc.cgi?acc=GSE117735], GSE121755 [https://www.ncbi.nlm.nih.gov/geo/query/acc.cgi?acc=GSE121755], GSE144843 [https://www.ncbi.nlm.nih.gov/geo/query/acc.cgi?acc=GSE144843]. All Raw data of GC-MS can be found in metabolomics workbench75 using the Study ID ST002578 [https://www.metabolomicsworkbench.org/data/DRCCMetadata.php?Mode=Study&StudyID=ST002578&StudyType=MS&ResultType=1], ST002586 [https://www.metabolomicsworkbench.org/data/DRCCMetadata.php?Mode=Study&StudyID=ST002586&StudyType=MS&ResultType=1], and ST002587 [https://www.metabolomicsworkbench.org/data/DRCCMetadata.php?Mode=Study&StudyID=ST002587&StudyType=MS&ResultType=1]. All other data are included in main and supplemental figures and tables.

Human research participants

Policy information about studies involving human research participants and Sex and Gender in Research.

Reporting on sex and gender	Sex/gender, racial and ethnic considerations: SCCOHT is an ovarian disease of women andthus all cell line models, and patient-derived materials are all female origin. Ovarian cancer cell lines with intact SMARCA4 were all female origin, which were included as controls in our study. For lung cancers, as SMARCA4/2-deficiency is rare, all available cell lines and data from all patient tumors available from TCGA, cBioportal and DepMap database, regardless of their sex, gender, racial and ethnic groups, are included in our study.
Population characteristics	Small cell carcinoma of ovary, hypercalcemic type (SCCOHT) patient tumors - SCCOHT is a very rare diagnosis. It is so rare that there is no specific entry in the IARC book “Cancer Incidence in Five Continents”, but this cancer is included in “other” ovarian tumors. The rates for ‘other” ovarian tumors are 0.3 per 100,000 women for USA (Whites and Blacks), 0.2 in Canada, 0.3 in China, 0.1 in India, 0.2 in Germany and 0.1 in Australia. Thus there is very little international variance, and almost all cases have a single genetic cause (SMARCA4 pathogenic variants). These cases which are from all over the world (mostly US, Canada, Europe, Australia). The average diagnosis age of the SCCOHT patients in this study is 24.8 years; all patients are women; 15 Stage I, 7 Stage II, 13 stage III, 2 Stage IV, 47 not available (NA); treatment - 25 chemotherapy, 8 chemotherapy + radiation therapy (RT), 1 high dose chemotherapy (HDC), 1 chemotherapy + RT + HDC, 46 NA.<br><br>High grade serous ovarian cancer (HGSOC) patient tumors - 2 FIGO stage I, 3 stage II, 26 stage III, 1 stage IV ; the average age is 58 years. Low grade serous ovarian cancer (LGSOC) patient tumors- 4 stage I, 3 stage II, 16 stage III, 3 stage IV and 11 not available; the average age is 56 years. Endometrioid ovarian cancer (ENOC) patient tumors - 8 stage I, 2 stage II, 3 stage III, 1 stage IV and 4 not available; the average age is 58 years. Clear cell ovarian cancer (CCOC) patient tumors - 7 stage I, 5 stage II, 7 stage III, 2 stage IV; the average age is 58 years. All these epithelial ovarian cancer patients are women; chemotherapy used in first line of treatment consisted of standard carboplatin and paclitaxel.
Recruitment	SCCOHT- Because of this low incidence, we reached out to colleagues who had reported cases, or received cases where our opinion had been requested. There is no pre-selection for these cases, which are from all over the world (mostly US, Canada, Europe, Australia). HGSOC, LGSOC, CCOC, ENOC - All consecutive patients undergoing surgery for gynecologic cancer in the division of gynecologic oncology at Vancouver General Hospital. We do not see any self-selection bias or other biases that might affect the results.
Ethics oversight	Studies on SCCOHT patient tumors were approved by the Institutional Review Board (IRB) at McGill University, McGill IRB # A08-M61-09B. Studies on SCCOHT and all other ovarian cancer tumors were approved by IRB at the University of British Columbia (IRB # 18-01652).

Note that full information on the approval of the study protocol must also be provided in the manuscript.

Field-specific reporting

Please select the one below that is the best fit for your research. If you are not sure, read the appropriate sections before making your selection.

☒ Life sciences ☐ Behavioural & social sciences ☐ Ecological, evolutionary & environmental sciences

For a reference copy of the document with all sections, see nature.com/documents/nr-reporting-summary-flat.pdf

Life sciences study design

All studies must disclose on these points even when the disclosure is negative.

Sample size	Sample sizes for in vitro experiments (three to six replicates confirmed via independent replication studies) were chosen based on the standard practices of the field. For public data set analyses, sample sizes were determined based on what was available publicaly, authors had no influence over how sample sizes were chosen for the design of these studies. Each in vivo study, 4-8 tumors per group were used to have appropriate statistical power based on previous experience.
Data exclusions	No data was excluded.
Replication	The findings were reliably reproduced within Sidong Huang's lab or Yemin Wang's lab. For all experiments, there are at least two independent biological repeats and multiple technical repeats in each. In all instances, all the attempts at replicating the experiments produced similar results.
Randomization	Mice were randomized into 2 or 4 different groups, 4-8 mice for each group. When tumors reached a volume of approximately 100mm3, mice were enrolled for receiving either vehicle or indicated treatments.
Blinding	The person administering the drug or placebo was not blinded to the drug condition due to the complexity of experiments and limited personnel. However, the subsequent measurements were blinded to the treatment information.

Reporting for specific materials, systems and methods

We require information from authors about some types of materials, experimental systems and methods used in many studies. Here, indicate whether each material, system or method listed is relevant to your study. If you are not sure if a list item applies to your research, read the appropriate section before selecting a response.

Materials & experimental systems

n/a	Involved in the study	
☐	☒	Antibodies
☐	☒	Eukaryotic cell lines
☒	☐	Palaeontology and archaeology
☐	☒	Animals and other organisms
☒	☐	Clinical data
☒	☐	Dual use research of concern

Methods

n/a	Involved in the study	
☒	☐	ChIP-seq
☒	☐	Flow cytometry
☒	☐	MRI-based neuroimaging

Antibodies

Antibodies used	Methods, Compounds and antibodies<br>Antibodies against HSP90 (H-114, 1:1,0000) and β-Actin (Cat# sc-47778, 1:1,0000) were from Santa Cruz Biotechnology (Dallas, TX, USA); antibodies against cleaved PARP (Cat# 5625, 1:1,000), cleaved caspase-3 (Cat# 9664, 1:1,000) and SMARCA2 (Cat# 11996, 1:1,000) and SMARCA2 (clone HPA029981; Sigma, St Louis, MO, USA) were from Cell Signaling (Danvers, MA, USA); antibody against SMARCA4 (A300-813A, 1:1,000) and (ab110641, 1:5000) were from Bethyl Laboratories and Abcam, respectively; antibody against SLC2A1 (ab15309, 1:1,000) was from Abcam; antibody against GLUT1 (for IHC, 1:3000, ab115730) was from Abcam; Cyclin D1 (1:400, #2978) was from Cell Signaling Technology. antibody against SLC38A2 (BMP081, 1:1,000) was from MBL.<br>Secondary Antibodies: Antibody against Rabbit IgG (Cat# 1706515) from BioRad, Antibody against Mouse IgG (Cat# 1706516) from BioRad.
Validation	HSP90 - Santa Cruz sc-13119 validation stated on supplier's website https://www.scbt.com/p/hsp-90alpha-beta-antibody-f-8<br>β-Actin - Santa Cruz sc-47778 validation stated on supplier's website https://www.scbt.com/p/beta-actin-antibody-c4<br>SMARCA2 - Cell Signaling # 11996 validation stated on supplier's website https://www.cellsignal.com/products/primary-antibodies/brm-d9e8b-xp-rabbit-mab/11966<br>SMARCA2 - Sigma HPA029981validation stated on supplier's https://www.sigmaaldrich.com/CA/en/product/sigma/hpa029981?gclid=Cj0KCQiA14WdBhD8ARIsANao07hm5xo1BWZsGjYvvxq-V_CSHqA3x3zU_tEYuDVuNT4Bzc-CAmSHaxgaAiyBEALw_wcB&gclsrc=aw.ds<br>cleaved PARP - Cell Signaling #5625 validation stated on supplier's website https://www.cellsignal.com/products/primary-antibodies/cleaved-parp-asp214-d64e10-xp-rabbit-mab/5625<br>cleaved caspase-3 - Cell Signaling # 9664 validation stated on supplier's website https://www.cellsignal.com/products/primary-antibodies/cleaved-caspase-3-asp175-5a1e-rabbit-mab/9664<br>SMARCA4 - bethyl A300-813A validation stated on supplier's website https://www.bethyl.com/product/A300-813A/BRG1+SMARCA4+Antibody<br>SMARCA4 -Abcam ab110641 validation stated on supplier's website https://www.abcam.com/brg1-antibody-epncir111a-ab110641.html<br>SLC2A1 -Abcam ab15309 validation stated on supplier's website https://www.abcam.com/glucose-transporter-glut1-antibody-ab15309.html

GLUT1 (for IHC) -Abcam, ab115730 validation stated on supplier's website https://www.abcam.com/products/primary-antibodies/glucose-transporter-glut1-antibody-epr3915-ab115730.html
Cyclin D1 Cell Signaling Technology, #2978 validation stated on supplier's website https://www.cellsignal.com/products/primary-antibodies/cyclin-d1-92g2-rabbit-mab/2978
SLC38A2 -MBL BMP081 validation stated on supplier's website https://www.mblintl.com/products/bmp081/

Eukaryotic cell lines

Policy information about cell lines and Sex and Gender in Research

Cell line source(s)	OVCAR4: Dr. E. Wang (University of Calgary, Calgary, originally from NCI); BIN-67: Dr. B. Vanderhyden (Ottawa Hospital Research Institute, Ottawa) originally from Dr. S.R. Goldring (Hospital for Special Surgery, New York, originally derived from patients with ovarian carcinoma treated at the Dana-Farber Cancer Institute (Boston, MA)); SCCOHT-1:Dr. R. Hass (Medical University Hannover, Hannover, generated by Dr. R. Hass); PC9:Dr. R. Bernards (Netherlands Cancer Institute, Amsterdam, originally from Immuno-Biological Laboratories (IBL), Tokyo, Japan); OVCAR8, SKOV3: Dr. M. Witcher (McGill University, Montreal, originally from ATCC); IOSE80: Dr. N. Auersperg (The University of British Columbia, Vancouver)FT237, FT190: Dr. T.G. Shepherd (The Mary & John Knight Translational Ovarian Cancer Research Unit, Ontario, FT190 was originally provided by R. Drapkin, University of Pennsylvania, Philadelphia, PA); 293T: ATCC, CRL-3216; H1703: ATCC, CRL-5889; H1299: ATCC, CRL-5803; H1437: ATCC, CRL-5872; HCC827: ATCC, CRL-2868; A549: ATCC, CCL-185; H2030: ATCC,CRL-5914; H1568: ATCC,CRL-5876; H661: ATCC,HTB-183; H23: ATCC, CRL-5800; A427: ATCC, HTB-53; H522: ATCC, CRL-5810; H358: ATCC, CRL-5807; H441: ATCC, HTB-174; H1792: ATCC, CRL-5895; H1573: ATCC, CRL-5877; COV434: Sigma, 07071909;<br><br>TOV-112D: Anne-Marie Mes-Masson (Centre de recherche CHUM et; Institut du cancer de Montréal)<br>CaoV3: Dr. Nelly Auersperg (University of British Columbia, Vancouver, originally from Jorgen Fogh, MSKCC)<br>OVCAR3, OVCAR4 and OVCAR5: Dr. Nelly Auersperg (University of British Columbia, Vancouver, originally from Thomas C Hamilton, Fox Chase Cancer Centre)
Authentication	All cell lines used were authenticated with STR-GenePrinter
Mycoplasma contamination	All cell line were tested and mycoplasma free.
Commonly misidentified lines (See ICLAC register)	No commonly misidentified cell lines were used in the study.

Animals and other research organisms

Policy information about studies involving animals; ARRIVE guidelines recommended for reporting animal research, and Sex and Gender in Research

Laboratory animals	8–12-week-old in house bred female NOD.Cg-Prkdcscid Il2rgtm1Wjl/SzJ (NSG) mice. 7-9 week-old in house bred female NRG (NOD.Rag1KO.IL2RγcKO) mice.<br>Housing conditions:<br>Temp: 16 degrees min - 24 degrees max<br>Humidity: 15% low - 60% high<br>Photoperiod: 7am-7pm light, 7pm-7am dark (McGill); 6a-6pm light, 6pm-6am dark (UBC)<br>60 air exchanges per hour<br>Top filter: Allentown Polysulfone microbarrier tops with Remay filter paper<br>Corncob bedding brand: Bulk Tote Corncob Bedding<br>Supplier: Envigo RMS (Canada) Limited<br>Food:<br>Commercial name: Global soy protein-free, irradiated<br>Brand: Teklad Cat # 2020SX Supplier: Envigo RMS (Canada) Limited<br>Water:<br>Reverse Osmosis, Chlorinated<br>Shredded paper commercial name: Fibercore, Brand: Envirodri (25lbs bag), Supplier: Cedarlane Laboratories Ltd.
Wild animals	This study did not involve wild animals.
Reporting on sex	Xenograft studies of SCCOHT were performed in female mice to reflect the disease origin. The PDX model of SMARCA4/2-deficient lung cancer used in this study was the only model we can identify, which was derived from a female patient. Thus the xenograft experiment on this lung cancer model was conducted in female mice too.
Field-collected samples	This study did not involve samples collected from the field.
Ethics oversight	Animal experiments were carried-out according to standards outlined in the Canadian Council on Animal Care Standards (CCAC) and the Animals for Research Act, R.S.O. 1990, Chapter c. A.22, and by following internationally recognized guidelines on animal welfare. All animal procedures (Animal Use Protocol) were approved by the Institutional Animal Care Committee according to guidelines of the Canadian Council of Animal Care. All animal experiments were carried-out at the Goodman Cancer Research Center of McGill University and British Columbia Cancer Research Institute of University of British Columbia.

Note that full information on the approval of the study protocol must also be provided in the manuscript.
