# Supplementary material for: Alanine supplementation exploits glutamine dependency induced by SMARCA4/2-loss
Source: Nat Commun. 2023 May 20;14:2894. doi: 10.1038/s41467-023-38594-3 (PMC10199906; doi:10.1038/s41467-023-38594-3)
Supplement: Supplementary file 7 — Description of Additional Supplementary Files [file 41467_2023_38594_MOESM7_ESM.pdf]

Title: Supplementary Data 1.
Description: Differential dependency of genes between SMARCA4/2-dual deficient versus proficient cell lines.

Title: Supplementary Data 2.
Description: Pathway enrichment analysis of the top essential genes.

Title: Supplementary Data 3.
Description: Differentially expressed glycolysis and glucose metabolism-related genes between SMARCA4/2-dual deficient versus proficient cell lines.

Title: Supplementary Data 4.
Description: Differentially expressed glycolysis and glucose metabolism-related genes in ovarian and lung cancer cell lines.
