# Supplementary material for: Alanine supplementation exploits glutamine dependency induced by SMARCA4/2-loss
Source: Nat Commun. 2023 May 20;14:2894. doi: 10.1038/s41467-023-38594-3 (PMC10199906; doi:10.1038/s41467-023-38594-3)
Supplement: Supplementary file 8 — Peer Review File [file 41467_2023_38594_MOESM8_ESM.pdf]

Editorial Note: This manuscript has been previously reviewed at another journal that is not operating a transparent peer review scheme. This document only contains reviewer comments and rebuttal letters for versions considered at *Nature Communications*. Mentions of prior referee reports have been redacted.
